# Tumor‐derived proliferative CTCs and CTC clusters predict aggressiveness and early recurrence in hepatocellular carcinoma patients

**DOI:** 10.1002/cam4.5946

**Published:** 2023-06-19

**Authors:** Lina Zhao, Jinge Song, Yulin Sun, Qiang Ju, Hong Mu, Xiu Dong, Jing Ding, Yunhe Liu, Xuebing Wang, Liying Sun, Jianxiong Wu, Yuchen Jiao, Shichun Lu, Xiaohang Zhao

**Affiliations:** ^1^ State Key Laboratory of Molecular Oncology National Cancer Center/National Clinical Research Center for Cancer/Cancer Hospital, Chinese Academy of Medical Sciences and Peking Union Medical College Beijing China; ^2^ Medical Research Center, Peking Union Medical College Hospital Peking Union Medical College and Chinese Academy of Medical Sciences Beijing China; ^3^ Department of Hepatobiliary Surgery and You‐an liver Transplant Center Beijing You‐An Hospital, Capital Medical University Beijing China; ^4^ Department of Hepatobiliary Surgery National Cancer Center/National Clinical Research Center for Cancer/Cancer Hospital, Chinese Academy of Medical Sciences and Peking Union Medical College Beijing China; ^5^ Department of General Surgery Beijing Friendship Hospital, Capital Medical University Beijing China; ^6^ Liver Transplantation Center, National Clinical Research Center for Digestive Diseases (NCRC‐DD) Beijing Friendship Hospital, Capital Medical University Beijing China; ^7^ Department of Hepatobiliary Surgery The First Medical Center, Chinese PLA General Hospital Beijing China

**Keywords:** circulating tumor cells, CTC clusters, driver gene mutation, hepatocellular carcinoma, proliferative CTC percentage, recurrence‐free survival

## Abstract

**Background:**

Circulating tumor cells (CTCs), an indispensable liquid biopsy classifier, can provide extra information for the diagnosis and prognosis of hepatocellular carcinoma (HCC).

**Methods:**

We systematically analyzed the peripheral blood of preoperative HCC patients (*n* = 270) for CTC number, Ki67 index reflecting the proliferative CTC percentage (PCP), and CTC clusters correlated with the characteristics of malignant HCC tumors.

**Results:**

Driver gene mutations were found with high levels of consistency between CTCs and primary tumors (*n* = 73). CTC number and PCP were associated with tumor size, microvascular invasion (MVI), presence or absence of multiple tumors, and thrombosis significantly. CTC number and PCP robustly separated patients with and without relapse, with a sensitivity of 88.89%/81.48% and a specificity of 72.73%/94.81% in the training set (*n* = 104) and corresponding values of 80.00%/86.67% and 78.38%/89.19% in the validation set (*n* = 52), showing a better performance than that associated with the alpha‐fetoprotein (AFP) level. CTC number, PCP, CTC clusters, and MVI were independent significant risk factors for HCC recurrence (*P* = 0.0375, *P* < 0.0001, *P* = 0.0017, and *P* = 0.0157). A nomogram model based on these risk factors showed a considerable prediction ability for HCC recurrence (area under the curve = 0.947). PCP (training: log‐rank *P* < 0.0001; hazard ratio [HR] 30.13, 95% confidence interval [CI] = 11.12–81.62; validation: log‐rank *P* < 0.0001; HR 25.73, 95% CI = 5.28–106.60) and CTC clusters (training: log‐rank *P* < 0.0001; HR 17.34, 95% CI = 7.46–40.30; validation: log‐rank *P* < 0.0001; HR 9.92, 95% CI = 2.55–38.58) were more significantly correlated with worse recurrence‐free survival than CTC number (training: log‐rank *P* < 0.0001; HR 14.93, 95% CI = 4.48–49.78; validation: log‐rank *P* = 0.0007; HR 9.03, 95% CI = 2.53–32.24).

**Conclusion:**

PCP and CTC clusters may predict HCC recurrence and improve the performance of the serum biomarker AFP.

## Graphical abstract

Tumor‐derived proliferative CTC and CTC clusters predict aggressiveness and early recurrence in hepatocellular carcinoma. Proliferative CTC and CTC clusters may predict HCC recurrence and improve the performance of serum biomarker alpha‐fetoprotein.

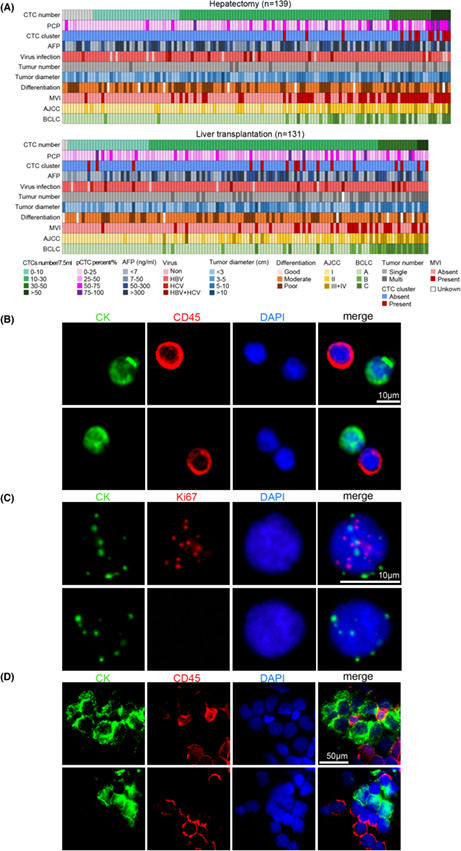

## 1 INTRODUCTION

Hepatocellular carcinoma (HCC) accounts for 75%–85% of primary liver cancers.^1^ Liver cancer was the third most common cause of cancer‐related death worldwide in 2020 and the second leading cause of cancer‐related death in China.^1^, ^2^ An estimated 905,000 new liver cancer cases and 830,000 deaths were reported worldwide in 2020.^1^ The pathogenesis of HCC is related to geographic location and ethnicity.^3^, ^4^

Although hepatectomy and liver transplantation are recognized as standard therapeutic practices for early‐stage HCC, a high risk of tumor recurrence still exists, with 70% and 35% of patients relapsing within 2 years after these surgeries, respectively.^5^, ^6^, ^7^, ^8^, ^9^ This information implies that the universally applied Barcelona Clinic Liver Cancer (BCLC) and American Joint Committee on Cancer (AJCC) staging systems are not effective for prognostic evaluations. In addition, the preoperative clinical diagnosis and prognostic evaluations of tumors are mainly based on imaging data and serum tumor marker levels. Traditional imaging methods are not sufficiently precise in diagnosing and staging HCC and underestimate the malignancy of the disease in some situations.^10^ Although alpha fetoprotein (AFP) remains the most widely used and accepted serum biomarker for HCC detection and monitoring, this marker is limited due to its low sensitivity.^11^, ^12^

Based on multiomics analysis of tumor specimens, stratification standards have been established to distinguish the malignant subtypes of HCC, even in the early clinical stage.^13^, ^14^, ^15^, ^16^ However, this strategy is limited by the sample source, its invasive nature, and tumor heterogeneity.^17^, ^18^ Thus, innovative blood tests for identifying individuals at risk of relapse are needed.

Liquid biopsy, including assessments of circulating tumor cells (CTCs), cell‐free DNA and cell‐free RNA, and vesicles (such as exosomes), is a revolutionary technique that can address the flaws in current diagnostic methods.^19^ This technology can mirror the features of the genomic, transcriptomic, and proteomic profiles of the primary tumor and serve as a reference for the early diagnosis of niduses, treatment response monitoring, and tumor recurrence prediction.

As one of the vital tumor markers of liquid biopsy, CTCs are a type of tumor cell with high activity and metastatic potential in the peripheral blood of solid tumor patients. The number and phenotype of CTCs are related to the progression of primary tumor lesions and can help elucidate the nature of the tumor lesions.^20^ After escaping from the host immune system, CTCs can lead to micrometastasis or microtumor thrombolysis. After entering the circulatory system, CTCs can disseminate to distant organs and form metastatic foci, which is a committed step in metastasis.^21^ CTCs have the capacity to proactively enter the circulatory system, and they contain the same genomic and transcriptional variations as primary and metastatic tumor cells in addition to unique variations and the ability to reflect genomic profiles during tumor evolution.^22^, ^23^ Therefore, these cells can effectively indicate tumor progression, metastasis, recurrence, and prognosis and have reference value for treatment selection.^24^, ^25^

CTCs originating from different tumor sources have high heterogeneity and are characterized by different surface markers and are different sizes. With the development of new identification techniques, more CTC phenotypes have been revealed. Metastasis‐related mesenchymal‐like CTCs are associated with poor overall survival and have spatial heterogeneity in the circulatory system.^26^, ^27^, ^28^, ^29^ Stem‐like CTCs have stronger drug resistance and anti‐apoptosis ability, and compared with regular CTCs, these cells have stronger tumorigenesis and invasion abilities in vivo. Moreover, stem‐like CTCs can be used as a diagnostic marker for tumors and show improved sensitivity and specificity.^30^, ^31^, ^32^ Consisting of 2–50 tumor cells, CTC clusters have a much higher metastatic potential than single CTCs and are negatively correlated with progression‐free survival.^25^, ^33^, ^34^, ^35^

Ki67 is regarded as a proliferation index of the primary HCC in evaluations of tumor malignancies and is related to tumor size, high AFP level, and TNM stage. Ki67 is an important prognostic factor for the overall survival and progression‐free survival of patients with HCC.^36^, ^37^ CTCs with positive Ki67 expression have been shown to be associated with poor prognosis and metastasis in breast cancer.^38^, ^39^ Therefore, Ki67 serves as a biomarker for identifying active CTCs.

To date, no relevant study has explored the correlation between Ki67‐positive CTCs and poor prognosis of HCC. Herein, a cohort of 270 HCC patients was recruited and CTCs were isolated. Immunofluorescence (IF) and RNAscope were applied to identify and enumerate the CTC number, the Ki67‐positive proliferative CTC percentage (PCP) and CTC clusters. The preoperative CTC number and Ki67‐positive PCP are correlated with the characteristics of malignant HCC tumors, such as tumor diameter, tumor number, satellite nodules, thrombosis, microvascular invasion (MVI), and AJCC and BCLC stages. After performing verification in an independent sample set, we found that a CTC number >15/7.5 mL, a Ki67‐positive PCP > 29.6%, and CTC clusters could serve as HCC prognostic biomarkers. In addition, these factors were correlated with a worse recurrence‐free survival (RFS) rate and were independent significant risk factors for HCC recurrence. In addition, compared with the CTC number, the Ki67‐positive PCP and CTC clusters were better predictive biomarkers for the early recurrence of HCC.

## 2 MATERIALS AND METHODS

### 2.1 Patient characteristics and follow‐up

From October 2014 to June 2018, a total of 270 HCC patients from Cancer Hospital of the Chinese Academy of Medical Sciences, Beijing You‐An Hospital and Beijing Friendship Hospital were enrolled for CTC detection. The inclusion criteria included a pathologic or radiographic diagnosis of HCC. Tumor stage was determined according to the AJCC and BCLC staging systems. Tumor differentiation was defined according to the Edmondson grading system. Liver function score was measured by Child‐Pugh system. The clinical information of patients with HCC, such as age, sex, imaging data, biochemical results from liver function examinations, and pathological results, was collected. The clinical characteristics of the 270 HCC patients included in this study are summarized in Table S1. Patients were followed up with standard postoperative visits every 3 months to monitor for tumor recurrence. Follow‐up was terminated on July 30, 2020, and RFS was defined as the time interval between the date of the operation and the date of recurrence or the end of follow‐up. This study was carried out with the approval of the Institutional Review Board of the Ethics Committee of Cancer Hospital of Chinese Academy of Medical Sciences (ID: NCC3094 and 2021101216113802) and Beijing Friendship Hospital (ID: 1018‐768). Informed consent was obtained from participants and the study was performed in accordance with the guidelines of the Declaration of Helsinki.

### 2.2 Sample processing and CTC enrichment

Peripheral blood (7–10 mL) was collected from each participant in Streck tubes (Streck). First, peripheral blood mononuclear cells (PBMCs) were collected by density gradient centrifugation using histopaque‐1077 (Sigma–Aldrich). Second, both negative selection and positive selection strategies were used to enrich and isolate CTCs. For negative selection, the PBMCs were mixed with microbeads (Miltenyi Biotec) conjugated to anti‐human CD45 antibodies. CD45^+^ white blood cells were depleted by applying them to an LS column (Miltenyi Biotec, Bergisch Gladbach, Germany) with magnetic separation. The supernatants were transferred into a new tube and centrifuged at 1000*g* for 10 min, and unlabeled CD45− cells were spotted on glass slides for IF staining.^40^, ^41^ CTCs were defined as 4′,6‐diamidino‐2‐phenylindole (DAPI+)/CD45–/CK+, whereas white blood cells were defined as DAPI+/CD45+/CK– cells. A CTC cluster was defined as a cell mass containing more than two tumor cells (DAPI+/CD45–/CK+). The CTC number of each sample was calculated as the CTC count of each slide divided by the volume of the blood sample. The enriched CTCs were analyzed via Ki67 RNA in situ hybridization (ISH). For positive selection, CTCs were enriched using a CytoQuest microfluidic system (Abnova). After washing with RPMI medium, a buffy coat was gently and homothermally injected into an asialoglycoprotein receptor‐coated microfluidic slide (KA4573, Abnova). Then, the captured cells were stained with anti‐CK‐FITC, anti‐CD45‐PE, and DAPI. Cells were visualized under a fluorescence microscope (Nikon), and single CTC cells were collected for whole‐genome sequencing analysis.

### 2.3 IF staining

After fixation and air drying, the cells were subjected to IF staining with an antibody cocktail against pancytokeratin (CK8/18/19)‐FITC (Miltenyi, 130‐080‐101; 1:100 diluted in 2% BSA) and CD45‐PE (Miltenyi, 130‐045‐801; 1:300 diluted in 2% BSA) for 1 h at room temperature. The slide was washed three times with phosphate‐buffered saline. The nuclear dye DAPI (Sigma‐Aldrich) was applied, and stained cells were observed and recorded with a fluorescence microscope (Nikon).

### 2.4 RNA ISH

RNAscope ISH assays were performed on formalin‐fixed CTC slides. The RNAscope probes Homo sapiens‐Hs‐panCK‐C3 (404751‐C3) and Homo sapiens 2.5 LS Probe‐Hs‐MKI67‐C3 (591778‐C3) were synthesized by Advanced Cell Diagnostics. The location and expression level of the target RNA were detected by the RNAscope Multiplex Fluorescent Reagent Kit v2 (Advanced Cell Diagnostics, 320293) according to the manufacturer's instructions. In brief, the CTC slides were dehydrated in an ethanol gradient, treated with H_2_O_2_ for 10 min, and then treated with Protease Plus (Advanced Cell Diagnostics, 322000) at 40°C for 30 min. Next, the C3 probes were diluted in C1 probes at a 1:50 ratio for hybridization at 40°C for 2 h. Then, AMP1, AMP2, and AMP3 were added to the slides and incubated at 40°C for 30 min. After HRP treatment, C1 probes detected with TSA‐Plus FITC and C3 probes detected with TSA‐Plus Cy5 were added to the slides sequentially, with HRP blockers applied after every step. Finally, DAPI (Sigma‐Aldrich) was added to label the nuclei, and the slices were mounted. Proliferative CTCs were defined as DAPI+/Ki67+/CK+ cells, and regular CTCs were defined as DAPI+/Ki67–/CK+ cells.

### 2.5 Single‐cell whole‐genome amplification

The identified CTCs from the positive enrichment strategy were separated into phosphate buffer solution by a micromanipulator with a 10 μM capillary needle and stored at −80°C for genomic analyses. Single‐cell whole‐genome amplification of CTCs and quality control genomic DNA amplification were carried out using the REPLI‐g Single Cell Kit (Qiagen) according to the manufacturer's protocol. The CTC pool was sized up to 4 μL using phosphate buffer, 3 μL denaturant (DLB: DTT = 11:1) was added, and degeneration was performed for 10 min at 65°C. Then, 3 μL stop solution was added to terminate degeneration. After oscillation and blending, 29 μL of reaction buffer, 2 μL of Phi29 DNA polymerase, and 9 μL of H_2_O were added, and the reaction was promoted for 4 h at 30°C. Phi29 DNA polymerase had strand displacement activity with high replication fidelity. For quality control, the amplification products were used as the template for secondary amplification, in which, *GAPDH*, *TP53*, and *CTNNB1* primers were designed for the target segment, and the CTC pool with at least one positive result was used for subsequent sequencing.

### 2.6 Statistical analysis

Statistical analysis was conducted with R software (version 3.6.1, R Foundation for Statistical Computing, Vienna, Austria) and GraphPad Prism (version 9.0, GraphPad Software). All measurement data are presented as the median±standard deviation (median±SD), and the minimum and maximum values are shown in parentheses. Differences between two groups were determined by the Mann–Whitney rank test. Differences among more than two groups were assessed with analysis of variance followed by a multiple comparisons test. The “pROC” package was applied to establish the receiver operating characteristic (ROC) curve to evaluate the prognostic values of the CTC number and PCP. “Survival” and “survminer” were used to perform univariate and multivariate Cox regression analyses and visualization of the results. Before the log‐rank test for RFS, continuous parameters, including of the CTC number and PCP, were dichotomized by optimal cutoff values determined based on their ROC curves. The nomogram model was constructed based on the final multivariate Cox regression model using the “rms” package. The performance of the nomogram was assessed using a calibration curve with 1000 bootstrap resamples for internal validation to assess their predictive accuracies. Decision curve analysis (DCA) was conducted with the “rmda” package to evaluate the efficacy of the nomogram. Analysis of similarity (ANOSIM) was performed using the “vegan” R package, and mutation state dissimilarity in ANOSIM was calculated based on the Euclidean distance matrix. The “wilcoxsign_test” of the “coin” package was applied to conduct permutation testing with 1000 resamples. All statistical tests were two‐tailed, and *P* < 0.05 was considered significant (*P < 0.05, **P < 0.0.01, ***P < 0.001, and ****P < 0.0001).

## 3 RESULTS

### 3.1 Patient characteristics

**FIGURE 1:**
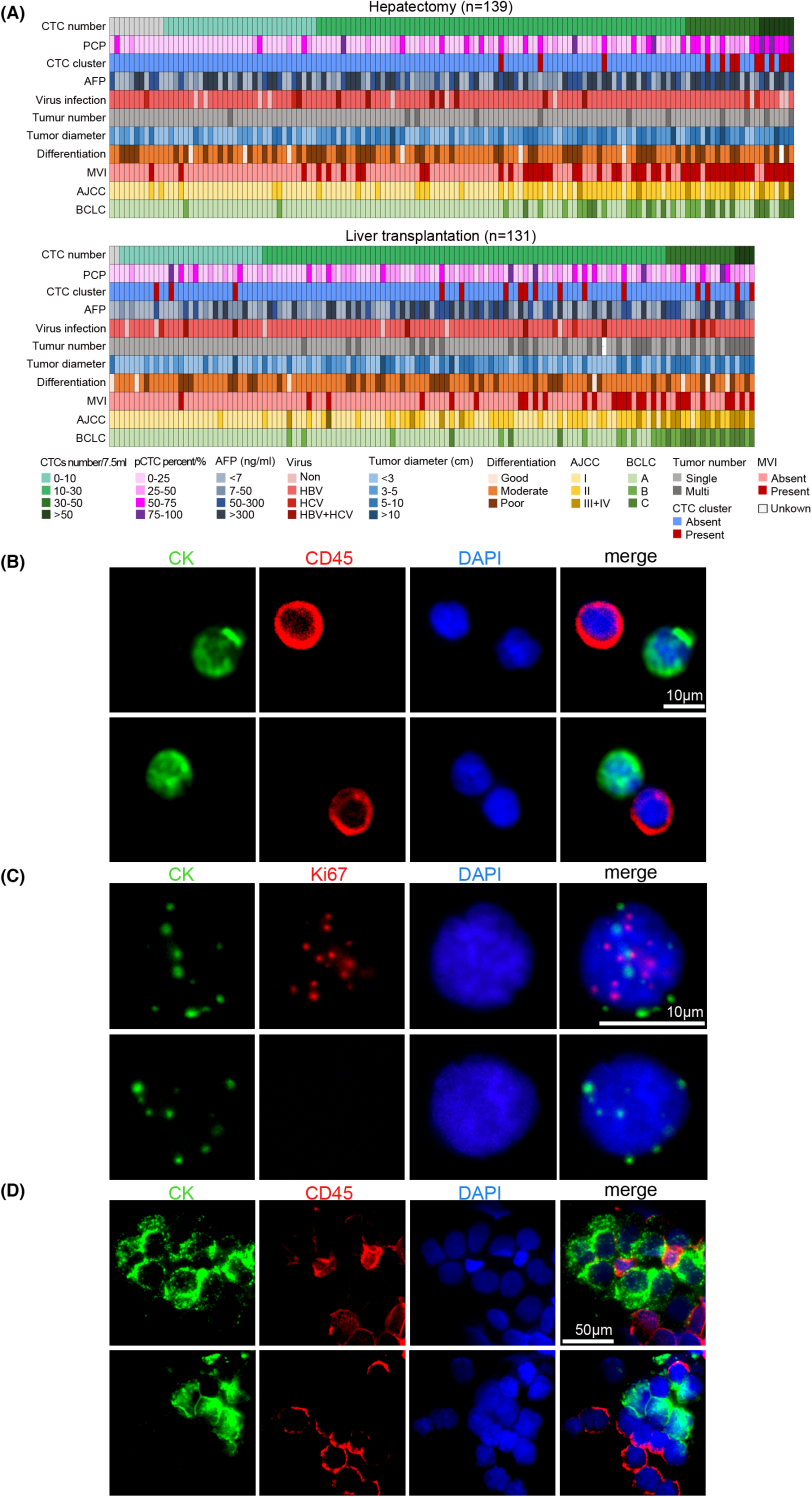
Identification of CTCs and proliferative CTCs. (A) Heatmap showing clinicopathological and CTC detection data; patients were stratified according to operation type. Basic clinical information is indicated by the colored bar above the heatmap. AJCC, American Joint Committee on Cancer; BCLC, Barcelona Clinic Liver Cancer; CTC, circulating tumor cell; MVI, microvascular invasion; PCP, proliferative CTC percentage. (B) CTCs were identified as DAPI+/CD45–/CK+ cells at 400× magnification. Scale bar: 10 μm. (C) Proliferative CTCs were defined as DAPI+/Ki67+/CK+ cells in the nucleus at 1000× magnification. Scale bar: 10 μm. (D) CTC clusters were identified as cell masses containing more than two tumor cells (DAPI+/CD45–/CK+ cells) at 400× magnification. Scale bar: 50 μm. CK, pancytokeratin‐FITC; DAPI, 4′,6‐diamidino‐2‐phenylindole.

A total of 270 patients with HCC from two hospitals were enrolled, and they underwent hepatectomy (*n* = 139) or liver transplantation (*n* = 131) surgery. The clinical information and pathological characteristics of the patients are summarized in Table S1 and Figure 1A. There were 228 males (84.44%) and 42 females (15.56%) with a median age of 55 (range 31–78); 91.11% (246/270) of the patients were hepatitis B virus positive, and 8.52% (23/270) were hepatitis C virus positive; 85.93% (232/270), 12.22% (33/270), and 1.85% (5/270) were classified as Child‐Pugh A, B, and C, respectively. In all, 83 patients (30.74%) received additional adjuvant therapy before surgery, including transcatheter arterial chemoembolization (13.33%, 36/270), radiofrequency ablation (7.78%, 21/270), and radiotherapy (3.96%, 8/270). The cohort was mainly composed of HCC patients with early‐stage disease. Specifically, 56.67% (153/270), 34.44% (93/270), 8.52% (23/270), and 0.37% (1/270) of patients were classified as AJCC stage I, II, III, and IV, and 76.67% (207/270), 12.59% (34/270), and 10.74% (29/270) were classified as BCLC stage A, B, and C, respectively (Table S1).

### 3.2 CTC identification and numeration

CTCs were identified as DAPI+/CD45–/CK+ cells and numerated in the preoperative peripheral blood of HCC patients. A total of 258 patients (95.56%) were found CTC positive (Figure 1B), and the median number of CTCs in HCC patients was 15 (range: 0–140) per 7.5 mL of blood (Figure 1A). Furthermore, Ki67 expression levels in CTCs were measured by RNAscope ISH and regarded as the proliferative index of CTCs. Approximately 80.37% (217/270) of HCC patients were CTC‐Ki67 positive based on the preoperative peripheral blood samples (Figure 1C). The percentage of DAPI+/Ki67+/CK+ CTCs was calculated and defined as the PCP. The median percentage of Ki67‐positive proliferative CTCs in the 270 HCC patients was 20% (range: 0%–92.88%). In addition, PCP was correlated with the positive Ki67 expression ratio in primary tumor cell in HCC (*n* = 76, person's *r* = 0.58, *P* < 0.0001; Figure S1A–C). Furthermore, approximately 10.74% (29/270) of patient samples were identified with CTC clusters (Figure 1D).

### 3.3 Mutation consistency between the primary tumor and CTCs

**FIGURE 2:**
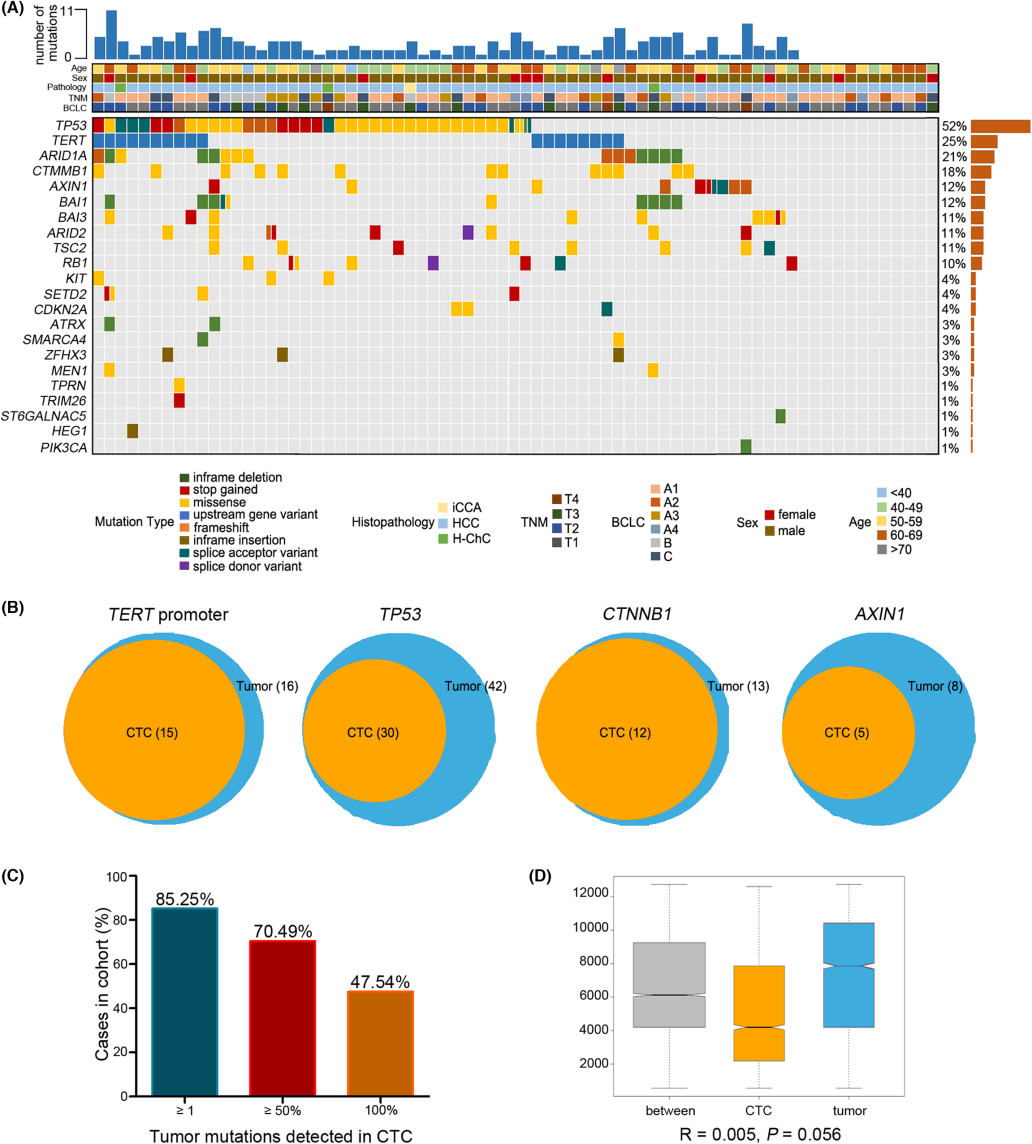
Consistency of mutations between primary tumor tissues and circulating tumor cells (CTCs). (A) Targeted sequencing of 16 driver genes was conducted in 73 HCC primary tumor tissues. (B) Venn diagrams manifesting the consistency of the *TERT* promoter, *TP53*, *CTNNB1*, and *AXIN1* mutations between tissues and CTCs. (C) The majority of cases show a high level of consistency among the driver gene variants detected in tumor tissues and corresponding CTCs in the same individuals. (D) The mutation consistency between the tumor tissues and CTCs is shown through analysis of similarities (ANOSIM), which was calculated based on the Euclidean distance matrix.

To verify whether the CTCs were tumor sourced, we further investigated the mutation consistency between the primary tumor tissues and CTCs. Coding regions in 16 driver genes were examined in tumor tissues (Table S2). The average depth (distinct coverage) of tumor tissue sequencing was 1535×. A total of 152 somatic SNVs and 52 somatic indels in a total of 61 tissue samples were identified by targeted sequencing in 73 HCC patients (Figure 2A; Table S3). The RFS of patients with driver gene mutations in tumor tissue was significantly different from that of patients without driver gene mutations in tumor tissues (log‐rank *P* = 0.0220; hazard ratio [HR] 7.31, 95% confidence interval [CI] = 0.99–53.75; Figure S2A). The median number of somatic mutations in coding regions was 2 (range 0–17). The genes with high mutation frequencies were *TP53* (*n* = 38, 52.05%), *TERT* promoter (*n* = 18, 24.66%), *ARID1A* (*n* = 15, 20.55%), *CTNNB1* (*n* = 13, 17.81%), and *AXIN1* (*n* = 9, 12.33%; Figure 2A; Table S3).

Then, the alterations discerned from primary tissue were verified in the corresponding CTCs by Sanger sequencing. Whole‐genome amplification was conducted by the multiple displacement amplification method, and those products with positive quality control were subjected to Sanger sequencing. We found that the mutation consistency of the four driver genes (*TERT* promoter, *TP53, CTNNB1*, and *AXIN1*) between the tumor tissues and the preoperative CTCs was 93.75% (15/16), 71.43% (30/42), 92.31% (12/13), and 62.50% (5/8), respectively, both were considerable (Figure 2B; Table S4).

Generally, for the CTC amplification‐positive patients (*n* = 58), 174 alterations were identified in the primary tumors, 107 (61.49%) of which overlapped with the homologous CTC pool. Among the patients with mutations in the tumor, 85.25% (52/61), 70.49% (43/61), and 47.54% (29/61) of them had at least one, half, and all identical mutations detected in the matched pool of whole‐genome amplification products of CTCs (Figure 2C). In addition, for the CTCs of patients with at least half of the driver gene mutations in tumor tissues, the RFS was worse than that of patients whose CTCs had less than 50% of the driver gene mutations detected in tumor tissues (median RFS for positive and negative groups, 21.9 months vs. not reached, log‐rank *P* = 0.0120; HR 2.74, 95% CI = 1.21–6.19; Figure S2B). For the four most consistent driver genes (*TERT* promoter, *TP53, CTNNB1*, and *AXIN1*), we found that the RFS of patients with *TP53* mutation in CTCs was significantly different from that of patients without *TP53* mutation in CTCs (median RFS for positive and negative groups, 21.4 months vs. not reached, log‐rank *P* = 0.0353; HR 2.19, 95% CI = 1.06–4.55; Figure S3). To measure examine the mutation status differences among the tumor tissues and CTCs, ANOSIM was carried out and further confirmed that there were no significant differences in mutation status between tumor tissues and CTCs (*P* = 0.0562; Figure 2D). These results confirmed that CTCs were tumor sourced and could reflect the mutation of the primary tumor.

### 3.4 Correlation of tumor malignancy with CTC number and PCP

**FIGURE 3:**
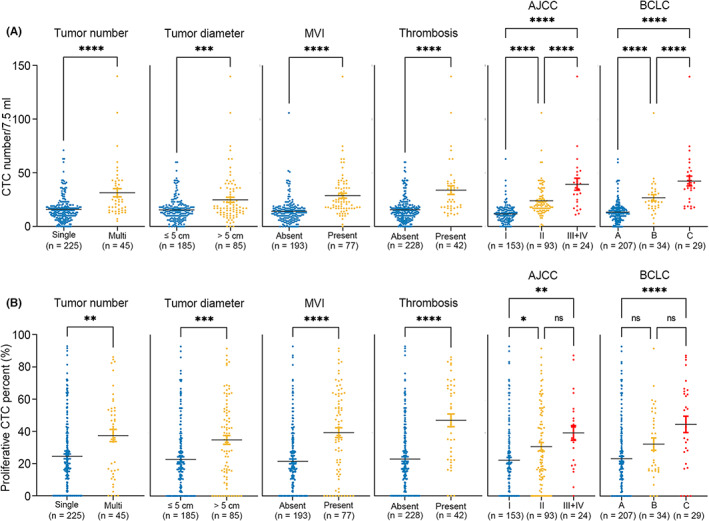
Correlations of CTC number and PCP with HCC prognosis‐related clinical parameters. (A) The scatter plots show the comparison of preoperative CTC numbers under different clinical stratification criteria, including single or multiple tumors, tumor diameter, MVI, thrombosis, AJCC stage, and BCLC stage. (B) The scatter plots show the comparison of preoperative PCP under different clinical stratification criteria, including single or multiple tumors, tumor diameter, MVI, thrombosis, AJCC stage, and BCLC stage (Mann–Whitney *t* test, two‐tailed and one‐way ANOVA test). AJCC, American Joint Committee on Cancer; BCLC, Barcelona Clinic Liver Cancer stage; CTC, circulating tumor cell; MVI, microvascular invasion; PCP, proliferative CTC percentage.

Subsequently, the correlations of the CTC number and PCP with the clinical parameters related to poor prognosis in HCC were evaluated. In this analysis, the CTC number in peripheral blood was associated with AFP level (*P* = 0.0041), tumor number (*P* < 0.0001), largest tumor diameter (*P* = 0.0003), MVI (*P* < 0.0001), thrombosis (*P* < 0.0001), AJCC stage (I vs. II, *P* < 0.0001; II vs. III, *P* < 0.0001; I vs. III + IV, *P* < 0.0001), and BCLC stage (A vs. B, *P* < 0.0001; B vs. C, *P* < 0.0001; B vs. C, *P* < 0.0001), indicating that CTC number was associated with HCC tumor burden and could be used to assess HCC progression (Figure 3A, Table S5).

Similarly, a higher PCP was found in patients with a high AFP level (*P* = 0.0135), multiple tumors (*P* = 0.0017), larger tumor diameter (*P* = 0.0002), MVI (*P* < 0.0001), thrombosis (*P* < 0.0001), AJCC stage (I vs. II, *P* = 0.0161; II vs. III, *P* = 0.2389; I vs. III + IV, *P* = 0.0017), and BCLC stage (A vs. B, *P* = 0.0786; B vs. C, *P* = 0.0830; B vs. C, *P* < 0.0001), revealing that the CTC number and PCP could be used to assess disease progression in HCC patients (Figure 3B, Table S5). In addition, neither the CTC number nor the PCP were correlated with other clinicopathological features (sex: *P* = 0.7685, *P* = 0.2162; age: *P* = 0.4780, *P* = 0.0766; differentiation: *P* = 0.8746, *P* = 0.8008; Table S5).

### 3.5 PCP was a more effective prognostic biomarker in HCC patients

The preoperative recurrence prediction values of the CTC number and PCP were assessed. A total of 156 HCC patients were ultimately involved in the follow‐up study after surgical therapy, and the other 114 cases were excluded from subsequent analysis due to lack of follow‐up. The median follow‐up time was 32.6 months (range, 25.6–63.8 months). During follow‐up, 42 patients (26.92%) exhibited disease recurrence confirmed by imaging, 39 patients underwent hepatectomy and exhibited a recurrence rate of 28.00% (35/125), and 7 patients underwent liver transplantation and exhibited a recurrence rate of 22.58% (7/31).

**FIGURE 4:**
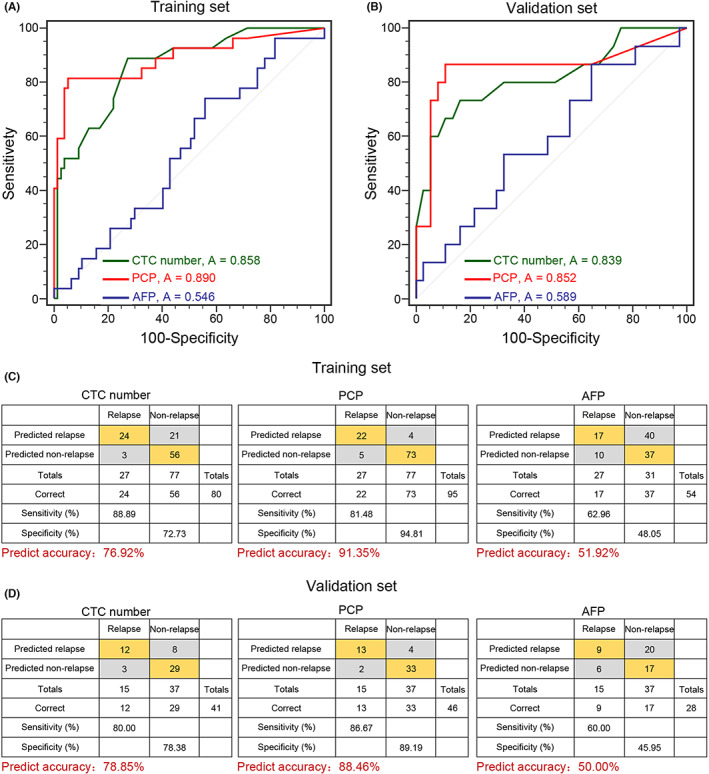
Performance of the CTC number and PCP in the training and validation data sets. (A) and (B) The capabilities of the CTC number, PCP and AFP level in predicting HCC recurrence were estimated with ROC curves in the training data set and validation data set. (C) and (D) Confusion tables of the binary results of the CTC number, proliferative CTCs, and AFP level in the training and validation data sets. A, area under the curve; AFP, alpha‐fetoprotein; CTC, circulating tumor cell; PCP, proliferative CTC percentage.

Initially, two‐thirds of the 156 HCC patients, a total of 104 HCC patients, namely, 27 relapsed patients and 77 non‐relapsed patients, were randomly selected as the training set. ROC curves were adopted to assess the ability of the CTC number, PCP and AFP level to predict postoperative recurrence, and all were noninvasive circulating biomarkers of HCC. The PCP and CTC number could be used to differentiate patients with recurrence (*n* = 27) from nonrecurrence patients (*n* = 77), with areas under the curve (AUCs) of 0.890 and 0.858, respectively, which were much higher than the AUC for AFP of 0.546 (Figure 4A). The PCP and CTC number had higher prediction accuracy and yielded sensitivities of 81.48% and 88.89% and specificities of 94.81% and 72.73%, respectively, and these values were better than those of AFP (positive defined as >20 ng/mL; sensitivity of 62.96% and specificity of 48.05%; Figure 4C). The cutoff values of the PCP and regular CTC number were 15/7.5 mL and 29.6%, respectively, which were the values with the highest Youden index.

Then, both circulating tumor markers were validated in the independent validation set of 52 HCC patients comprising 15 relapsed and 37 non‐relapsed HCC patients. Similarly, the AUCs of the PCP and CTC number were 0.852 and 0.839, respectively, which were still better than the AUC of 0.589 for AFP (Figure 4B). Based on the preoperative threshold originating from the training set, the PCP and CTC number could be used to discern 13 and 12 of 15 relapsed patients, respectively, and these results were confirmed by CT/MRI during follow‐up. In addition, 33 and 29 of 37 clinical patients without relapse were predicted to be negative by the PCP and CTC number, respectively. Thus, the PCP and CTC number yielded sensitivities of 86.67% and 80.00% and specificities of 89.19% and 78.36%, respectively, in the validation cohort, which were superior to those of AFP (sensitivity: 60.00%, specificity: 45.95%; Figure 4D). In addition, although CTC clusters showed average specificity in the ROC curve for recurrence prediction (Figure S4), their sensitivity for predicting recurrence reached 98.70% and 100% in the training and validation cohorts, respectively. These results indicated that the PCP and CTC number had consistent predictive abilities and even outperformed the serum AFP level. In particular, the PCP tended to precisely separate relapsed from non‐relapsed patients, which was also better than Ki67 expression ratio in primary tumor cells (AUC = 0.887 vs. AUC = 0.717; Figure S1D–F).

### 3.6 Correlation between the CTC number, the PCP, CTC clusters, and the prognosis of surgical treatment

**FIGURE 5:**
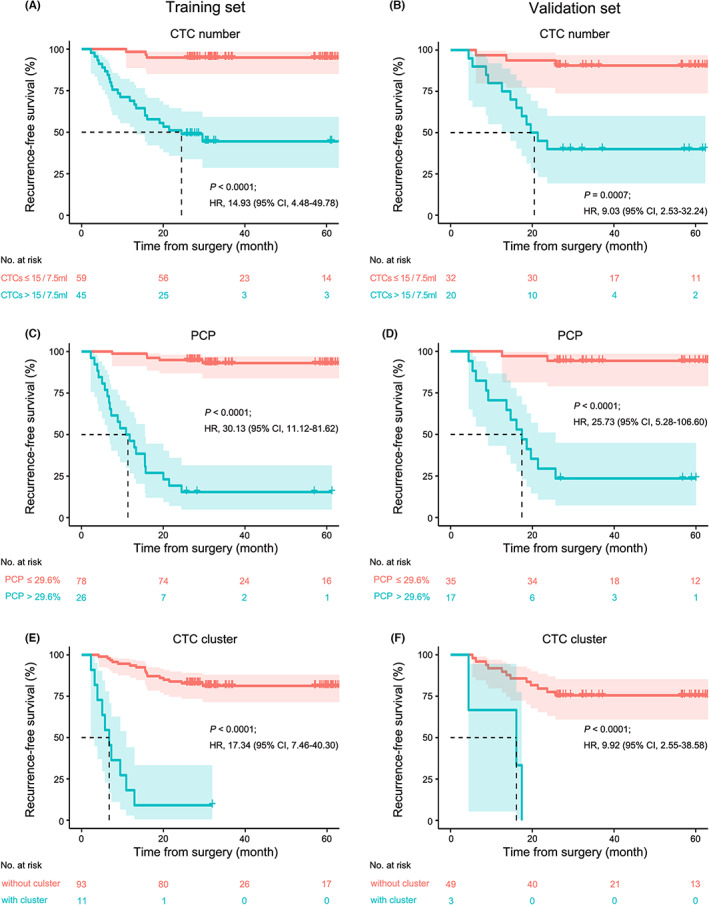
Performance of the CTC number, PCP and CTC clusters in predicting recurrence‐free survival. Kaplan–Meier analysis of (A) CTC number, (C) PCP, and (E) CTC clusters for predicting RFS in the training data set. Kaplan–Meier analysis of (B) CTC number, (D) PCP, and (F) CTC clusters for predicting RFS in the validation data set. The cutoff value and *P* values were derived from log‐rank (Mantel–Cox) tests, and HR values are indicated in the figure. The dashed lines in the figure represent the median survival (month). CI, confidence interval; CTC, circulating tumor cell; HR, hazard ratio; PCP, proliferative CTC percentage; RFS, recurrence‐free survival.

Furthermore, Kaplan–Meier analysis was conducted to evaluate whether the preoperative CTC number, CTC clusters, the PCP, and the AFP level could be used to evaluate the recurrence risk. The thresholds for a positive CTC number and PCP were defined based on their ROC curves. In all, 35 of the 68 (57.37%) patients with positive CTCs exhibited disease recurrence compared with 7 of the 88 (7.95%) patients with negative CTCs (Fisher's exact test, *P <* 0.0001). In all, 13 of 14 (92.85%) HCC patients with CTC clusters exhibited recurrence, which was more serious than that in 29 of 142 HCC patients without CTC clusters (Fisher's exact test, *P <* 0.0001). Similarly, 35 of the 43 (81.40%) patients with a positive PCP exhibited disease recurrence, compared with 7 of the 113 patients with a negative PCP (6.19%; Fisher's exact test, *P <* 0.0001). In addition, the RFS of patients with positive CTC numbers preoperatively was significantly different from that of patients with negative CTC numbers preoperatively in both the training data set and validation data set (median RFS for positive and negative groups, 24.5 months vs. not reached, log‐rank *P* < 0.0001; HR 14.93, 95% CI = 4.48–49.78; 23.7 months vs. not reached, log‐rank *P* = 0.0007; HR 9.03, 95% CI = 2.53–32.24; Figure 5A,B). Moreover, as the factor more related to recurrence, the PCP was significantly correlated with worse RFS in the training data set (11.4 months vs. not reached, log‐rank *P* < 0.0001; HR 30.13, 95% CI = 11.21–81.62) and validation data set (17.4 months vs. not reached, log‐rank *P* < 0.0001; HR 25.73, 95% CI = 5.28–106.60; Figure 5C,D). CTC clusters were also significantly correlated with worse RFS in the training data set (3.17 months vs. not reached, log‐rank *P* < 0.0001; HR 17.34, 95% CI = 7.46–40.30) and validation data set (16.1 months vs. not reached, log‐rank *P* < 0.0001; HR 9.92, 95% CI = 2.55–38.58; Figure 5E,F). No significant association was identified between AFP positivity at baseline (>20 ng/mL) and worse RFS in the training data set or validation data set (*P* = 0.3230, *P* = 0.6460; Figure S5A,B).

### 3.7 Risk factors and nomogram prediction model for early HCC recurrence

The independent risk factors for early HCC recurrence were investigated further. Multivariate Cox regression analysis using parameters that were significant in univariate analysis, including multiple nodules, thrombosis, MVI, tumor diameter, CTC number, CTC clusters, and PCP, revealed that the presence of MVI (HR, 3.00; 95% CI, 1.23–7.31; *P* = 0.0157), CTC number (HR, 2.96; 95% CI, 1.07–8.21; *P* = 0.0375), CTC cluster (HR, 3.79; 95% CI, 1.65–8.72; *P* = 0.0017), and PCP (HR, 13.18; 95% CI, 5.01–34.67; *P* < 0.0001) were significant independent risk factors for HCC recurrence, while multiple nodules, thrombosis, and BCLC stage were not (Table S6). These results suggested that the number and phenotype of CTCs could be warning markers of early recurrence of HCC.

**FIGURE 6:**
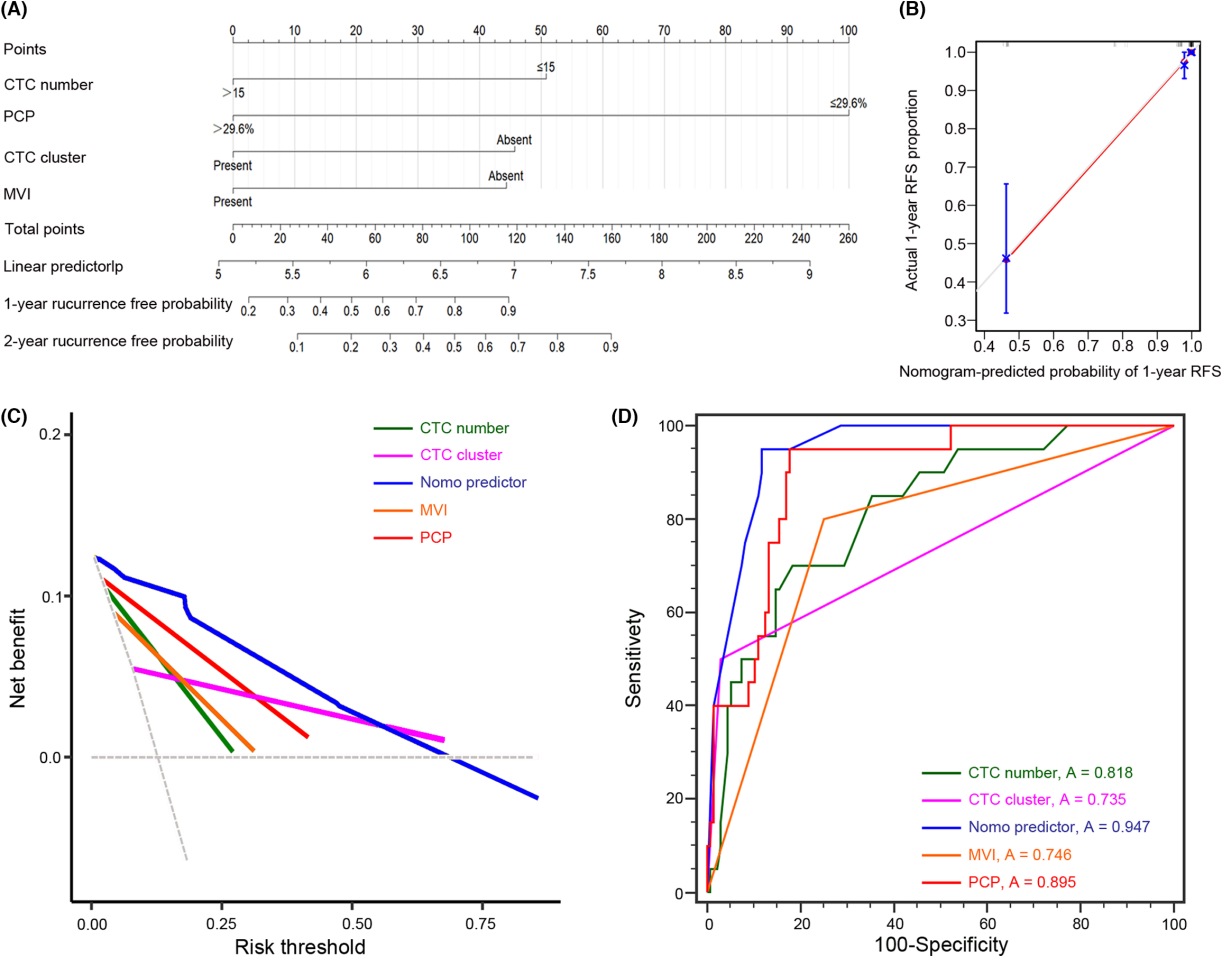
Establishment of recurrence‐free survival nomograms. (A) A nomogram predicting the risk of recurrence in patients with HCC. The value of each of variable was given a score on the point scale axis. A total score could be easily calculated by adding each single score, and by projecting the total score to the lower total point scale, the probability of recurrence was estimated. (B) The 1‐year calibration curves for the nomogram. The x‐axis represents the nomogram‐predicted probability, and the y‐axis represents the actual probability of 1‐year recurrence. (C) Decision curve analysis of the nomogram predictor, CTC number, PCP, CTC clusters, and MVI. (D) The capabilities of the nomogram predictor, CTC number, PCP, CTC clusters, and MVI in predicting HCC recurrence were estimated with ROC curves in the total data set. A, area under the curve; CTC, circulating tumor cell; MVI, microvascular invasion; PCP, proliferative CTC percentage; RFS, recurrence‐free survival.

On the basis of the multivariate Cox regression analysis, a nomogram was constructed that incorporated the four significant risk factors for predicting recurrence (Figure 6A). A total score was calculated with the use of MVI, CTC numbers, CTC clusters, and PCP. A calibration curve of the nomogram is presented in Figure 6B, which shows that the 1‐year recurrence probabilities predicted by the nomogram agreed well with the actual probabilities. Next, to further compare the nomograms with a single risk factor, DCA and ROC curve analysis were performed for all HCC patients. In DCA, nomograms showed superior power to MVI, CTC number, CTC clusters, and PCP (Figure 6C). Consistently, based on the ROC curve analysis, the nomogram showed a robust discrimination, with an AUC of 0.947, which was superior to those of MVI (AUC = 0.746), CTC number (AUC = 0.818), CTC clusters (AUC = 0.735), and PCP (AUC = 0.895; Figure 6D). The cutoff value of the nomogram was 2.67, with the highest Youden index for ROC curve analysis. In addition, nomograms for RFS showed higher statistical power (HR 34.96, 95% CI = 16.08–78.00) for CTC number (HR 11.89, 95% CI = 5.00–28.30), PCP (HR 26.88, 95% CI = 11.68–61.31), and CTC clusters (HR 13.67, 95% CI = 7.00–27.88; Figure S6). The results above showed that the nomogram prediction model can predict prognosis more accurately than CTCs alone.

## 4 DISCUSSION

The limitations of existing staging systems and serum markers for HCC are noteworthy, as evidenced by the high relapse rate following surgical interventions and the inefficiency in identifying and predicting patients prone to relapse. To compensate for these issues, this study explored the potential of the CTC number and phenotype to predict disease recurrence in a large cohort of HCC patients. We compared the abilities of the number/different subtypes of CTCs and serum biomarkers to predict tumor relapse in patients with HCC. AFP, as the most widely applied serum biomarker applied in HCC patient management, does not meet the clinical requirements, as demonstrated by its low sensitivity (only approximately 30%) in predicting tumor relapse and its weak associations with patient survival.^42^, ^43^ The diagnostic and prognostic efficacy of other serum protein biomarkers, such as AFP‐L3 and des‐γ‐carboxy, are only slightly higher than that of AFP.^44^, ^45^ Consistent with previous studies, the preoperative CTC number was correlated more closely with patient prognosis than serum biomarkers, especially AFP levels. Therefore, it is recommended for predicting tumor recurrence.^46^, ^47^ In addition, the Ki67‐positive proliferative CTC subtype was identified in HCC patients for the first time, and the predictive ability of this parameter for early recurrence was confirmed; notably, this variable was even more effective than the CTC number and comparable to CTC clusters in the same cohort. In addition, CTC‐related variables were especially helpful in AFP‐negative patients; 70 out of 156 HCC patients in the cohort (44.87%), 16 of whom had no warning biomarkers, exhibited tumor recurrence, but all of them could be identified by the CTC numbers or PCP. CTC‐based tumor recurrence prediction could provide earlier warning of recurrence, which may allow for prospective adjuvant treatment and increase the possibility of favorable outcomes.

CTCs are capable of vessel invasion and the formation of metastatic loci; thus, studies have focused on exploring the biological characteristics of CTCs from multiple dimensions.

The molecular characteristics of CTCs, which represent a state between primary and metastatic tumors, could assist in accurate classification, exploration of tumor cell subgroups with high malignancy, and targeted drug selection.^22^, ^23^, ^48^ Confirmed in 73 patients, the largest cohort of HCC CTC sequencing to date, our inquiry suggested that driver gene mutations in CTCs were highly consistent with those in the primary lesion.^49^ In addition, the mutation status of CTCs had significant heterogeneity, affirming that CTCs came from tumor tissue but might undergo a complex subclonal evolution. In summary, CTCs could act as the cornerstone for individualized treatment such as early diagnosis, dynamic monitoring, and detection of drug resistance variation, thus generating considerable potential clinical application prospects.

The CTC number could be used for early diagnosis, prognosis evaluation, and evaluations of response to antitumor drugs in patients with multiple tumors.^50^, ^51^ An increasing number of studies have concentrated on different phenotypes of CTCs and their potential clinical relevance. Correlated with disease progression, mesenchymal CTCs are more sensitive than epithelial CTCs in postoperative follow‐up^26^, ^27^, ^29^ The spatial heterogeneity of the epithelial and mesenchymal transition phenotype in CTCs within the circulatory system has been evaluated in HCC patients. CTCs from different vascular sites were subjected to single‐cell full‐length RNA sequencing to depict the transcriptional signature. The transcriptional dynamics of CTCs have been shown to be associated with the stress response, cell cycle, and immune evasion signaling during hematogenous transportation.^52^

Stem‐like CTCs have stronger drug resistance and anti‐apoptosis effects and contribute to tumorigenesis and metastasis by self‐seeding, which has been identified in animal models. In addition, stem‐like CTCs have been demonstrated in large cohorts to have more diagnostic value.^31^, ^32^ Small triploid CTCs are associated with poor prognosis.^30^ CTC clusters are composed of 2–50 CTCs, of which stem cell‐related markers (*OCT4, SOX2*, *Nanog*, and *SIN3A*) are hypermethylated. CTC clusters are associated with poor prognosis but are sensitive to some targeted drugs, such as Na+/K+‐ATPase inhibitors, affording a theoretical basis for precision therapy. It seems that neutrophils are involved in the formation of CTC clusters and promote the CTC cell cycle process to promote metastasis.^25^, ^34^ Our research verified that the preoperative CTC number was not only positively related to tumor progression, as demonstrated by AJCC stage, but also had a high sensitivity and specificity for predicting HCC relapse, indicating that the CTC number could be regarded as an HCC prognosis biomarker. Consistent with previous studies, HCC patients with CTC clusters had a worse prognosis after surgical intervention.^35^ Building on a previous study,^38^ proliferative CTCs were identified in our study through a more accurate strategy, RNAscope. The PCP was significantly correlated with poor prognosis and could be an indicator of HCC recurrence. In addition, the PCP and CTC clusters had an even better performance than the regular CTC number in predicting recurrence and RFS rate. Such results indicated that the more precise detection of CTCs may be more effective in clinical application.

Our research showed that tumor size (positive defined as >5 cm), AFP level (positive defined as >20 ng/mL), and BCLC staging (positive defined as >A stage), which are available preoperatively, were not effective in the prediction of postoperative relapse (sensitivity/specificity values of 50.00%/73.68%; 61.90%/47.36%; and 50.00%/86.84%, respectively). The remaining reference pathological parameters, such as MVI, thrombosis, and satellite nodules, depend on the postoperative surgical tumor specimens. However, the CTC number and PCP as well as liquid biopsy convey related information derived from the primary tumor; therefore, this strategy could more consistently predict relapse cases before surgery than other clinical biomarkers (sensitivity/specificity values of 83.33%/71.05% and 83.33%/92.98%). Although the detection rate of CTC clusters was only 10.74% in patients with HCC, the specificity of this parameter for predicting postoperative recurrence was as high as 98.61% (71/72) in the total cohort. In addition, a nomogram prediction model was constructed based on the independent risk factors for the CTC number, PCP, CTC clusters, and MVI in the multivariate analysis. As evaluated by the DCA, ROC curves, and survival curves, the comprehensive nomogram prediction model was proven to be more accurate in prognosis prediction.

These results illustrate that both the CTC number and specific phenotype could work as biomarkers of early recurrence in patients with HCC and help prolong survival and eventually reduce mortality. It has been reported that short‐term CTC culture could provide an opportunity to monitor drug susceptibility and response to adjuvant therapies.^53^ Reasonable adjuvant therapies for high‐risk recurrence HCC patients stratified based on CTCs need to be further confirmed by rigorous clinical research.

In summary, CK and CD45 staining were used to identify regular CTCs and CTC clusters in the peripheral blood of patients with HCC. Furthermore, Ki67 was used as a marker of proliferative CTCs to identify the most active subtype of CTCs. The preoperative CTC number, CTC clusters, and PCP were significantly associated with the RFS rate in HCC patients and regarded as independent significant risk factors for HCC recurrence. The PCP showed a better performance in predicting recurrence than the CTC number, and both were significantly better than the AFP level. Inevitably, there are some shortcomings in our study: (1) only two subtypes of CTCs were analyzed in this study, so the overall heterogeneous CTC situation in HCC patients may not be accurately illustrated, and the spatial heterogeneity of CTCs from different vascular sites was not discussed; (2) the number of cases used for the survival analysis was relatively small in this study; and (3) the molecular characteristics of CTCs, both at the genomic and transcriptomic levels, need to be further explored, which we aim to address in future studies. In conclusion, our study revealed that both the number and specific phenotype of CTCs could be used to support the clinical management of HCC patients and thus provide a foundation for future studies.

## AUTHOR CONTRIBUTIONS

**Lina Zhao:** Data curation (equal); formal analysis (equal); investigation (equal); methodology (equal); validation (equal); visualization (equal); writing – original draft (equal). **Jinge Song:** Data curation (equal); formal analysis (equal); investigation (equal). **Yulin Sun:** Data curation (equal); formal analysis (equal); funding acquisition (equal); investigation (equal). **Qiang Ju:** Data curation (equal); formal analysis (equal); investigation (equal); visualization (equal). **Hong Mu:** Data curation (equal); formal analysis (equal); investigation (equal); visualization (equal). **Xiu Dong:** Data curation (equal); formal analysis (equal); investigation (equal). **Jing Ding:** Data curation (equal); investigation (equal); resources (equal). **Yunhe Liu:** Data curation (equal); investigation (equal); resources (equal). **Xuebing Wang:** Data curation (equal); investigation (equal); resources (equal). **Liying Sun:** Data curation (equal); investigation (equal); resources (equal). **Jianxiong Wu:** Data curation (equal); investigation (equal); resources (equal). **Yuchen Jiao:** Data curation (equal); formal analysis (equal); funding acquisition (equal); methodology (equal); supervision (equal). **Shichun Lu:** Data curation (equal); formal analysis (equal); investigation (equal); resources (equal); supervision (equal). **Xiaohang Zhao:** Conceptualization (lead); funding acquisition (lead); project administration (lead); resources (equal); supervision (lead); writing – review and editing (equal).

## FUNDING INFORMATION

This work was partly supported by the National Key R & D Program of China (2017ZX10203205‐003‐001 and 2018YFC1313101 to XZ), the National Natural Science Foundation of China (81872033 to XZ and 82073327 to YS), and the CAMS Innovation Fund for Medical Sciences (2016‐I2M‐1‐001 and 2019‐I2M‐1‐003 to XZ; 2021‐I2M‐1‐066 to YS).

## CONFLICT OF INTEREST STATEMENT

The authors declare that they have no conflicts of interest.

## ETHICS STATEMENT

This study was carried out with the approval of the Institutional Review Board of the Ethics Committee of Cancer Hospital of Chinese Academy of Medical Sciences (ID: NCC3094 and 2021101216113802) and Beijing Friendship Hospital (ID: 1018‐768). Informed consent was obtained from participants in accordance with respective committee regulations and the study was performed in accordance with the guidelines of the Declaration of Helsinki.

## Supporting information


Table S1–S6

Figure S1–S6

## Data Availability

All data relevant to the study are included in the article or uploaded as online supplemental information. Additional data related to this paper may be requested from the corresponding authors.
