# Supplementary material for: Tumor‐derived proliferative CTCs and CTC clusters predict aggressiveness and early recurrence in hepatocellular carcinoma patients
Source: Cancer Med. 2023 Jun 19;12(13):13912–27. doi: 10.1002/cam4.5946 (PMC10358265; doi:10.1002/cam4.5946)
Supplement: Supplementary file 1 — Table S1–S6 [file CAM4-12-13912-s002.pdf]

Table S1. Clinical and Pathological Characteristics of HCC Patients

Clinicopathologic parameters		
Age (N = 270)	Median years 55 (31-78)	
Sex (N = 270)	Female	42 (15.56%)
	Male	228 (84.44%)
ALT (U/L) (N = 270)	Median 34.4(9–2342)	
AST (U/L) (N = 270)	Median 40.45 (11.7–7058)	
TBIL (umol/L) (N = 270)	Median 16.75 (4.2–488)	
ALP(U/L) (N = 270)	Median 72 (23.7–868)	
WBC(*10^9/L) (N = 270)	Median 5.84 (1.48–29.49)	
HGB(g/L) (N = 270)	Median 135.5(4.3–233)	
PLT(*10^9/L) (N = 270)	Median 137.5 (8–1136)	
AFP (ng/ml) (N = 270)	Median 33.78 (0.61–12100)	
Largest tumor diameter (mm) (N = 270)	Median 38 (3–210)	
Surgical procedure (N = 270)	Hepatectomy	139 (51.48%)
	Liver transplantation	131 (48.52%)
Multifocal (N = 270)	Absent	225 (83.33%)
	Present	45 (16.67%)
HBV (N = 270)	Absent	24 (8.89%)
	Present	246 (91.11%)
HCV (N = 270)	Absent	247 (91.48%)
	Present	23 (8.52%)
Prior treatment (N = 270)	Absent	187 (69.26%)
	TACE	36 (13.33%)
	RFA	21 (7.78%)
	Hepatectomy	22 (8.15%)
	RTx	8 (2.96%)
	Chemotherapy	1 (0.37%)
MVI (N = 270)	Absent	193 (71.48%)
	Present	77 (28.52%)
Tumor thrombosis (N = 270)	Absent	228 (84.44%)
	Present	42 (15.56%)
Differentiation grade (N = 268)	Well	13 (4.81%)
	Moderately	171 (63.33%)
	Poorly	84 (31.11%)
Child Pugh class (N = 270)	A	232 (85.93%)
	B	33 (12.22%)
	C	5 (1.85%)
	I	153 (56.67%)

Clinicopathologic parameters		
AJCC stage (N = 270)	II	93 (34.44%)
	III	24 (8.89%)
	A	207 (76.67%)
BCLC stage (N = 270)	B	34 (12.59%)
	C	29 (10.74%)

AFP, alphafetoprotein; HBV, hepatitis B virus; HCV, hepatitis C virus; AJCC, American Joint Committee on Cancer; BCLC, Barcelona clinic liver cancer stage; MVI, microvascular invasion; ALT: Alaninetransaminase; AST: Alaninetransaminase; TBIL: total bilirubin; ALP: alkaline phosphatase; WBC: white blood cell; HGB: hemoglobin; PLT: platelet count; TACE: transcatheter arterial chemoembolization; RFA: radiofrequency ablation; RTx: radiotherapy.

Table S2. Tumor tissue target sequencing panel

Target ID	Interval
ADGRB1	chr8:143545550-143625788
ADGRB3	chr6:69348558-70098793
ARID1A	chr1:27022885-27107257
ARID2	chr12:46123610-46298871
ATRX	chrX:76763819-77041497
AXIN1	chr16:338112-397035
CDKN2A	chr9:21968198-21994463
TERT promoter	chr5:1295000-1301000
CTNNB1	chr3:41265550-41280843
KIT	chr4:55524172-55604733
MEN1	chr11:64571796-64577591
RB1	chr13:48878039-49054217
SETD2	chr3:47058573-47205424
SMARCA4	chr19:11094818-11175887
TP53	chr17:7565247-7579922
TSC2	chr16:2098261-2138621

Table S3. Tumor mutations detected in target panel sequencing

Sample_ID	Gene_Name	Entrez_Gene_Id	Chromosome	Start_position	End_position	Variant_Classification	Variant_Type	Ref	Alt	dbSNP_RS	Exon_num	cDNA_Change	Protein_Change	Tumor_depth	Tumor_mutant_frequency
L100tumor	*AXIN1*	8312	16	396792	396792	frameshift_variant	DEL	G	-	.	2|11	c.234delC	p.Thr79ProfsTer5	3289	0.135
L100tumor	*ARID2*	196528	12	46245966	46245966	stop_gained	SNP	A	T	.	15|21	c.4060A>T	p.Arg1354Ter	939	0.15
L100tumor	*TSC2*	7249	16	2108755	2108755	missense_variant	SNP	A	G	rs1800748	10|42	c.856A>G	p.Met286Val	1308	0.504
L100tumor	*TSC2*	7249	16	2112569	2112569	missense_variant	SNP	G	T	.	13|42	c.1329G>T	p.Gln443His	1937	0.136
L100tumor	*TSC2*	7249	16	2121870	2121870	missense_variant	SNP	G	A	rs200494044	19|42	c.2032G>A	p.Ala678Thr	3910	0.479
L100tumor	*AXIN1*	8312	16	396815	396815	stop_gained	SNP	C	A	.	2|11	c.211G>T	p.Glu71Ter	1800	0.144
L100tumor	*PIK3R5*	23533	17	8792116	8792118	inframe_deletion	DEL	CCT	-	rs757971428	10|19	c.986_988delAGG	p.Glu329del	39	0.231
L102tumor	*TP53*	7157	17	7577548	7577548	missense_variant	SNP	C	T	.	7|11	c.733G>A	p.Gly245Ser	1515	0.424
L102tumor	*ARID1A*	8289	1	27100182	27100184	inframe_deletion	DEL	GCA	-	rs374564889	16|20	c.3999_4001delGCA	p.Gln1334del	4188	0.033
L102tumor	*ARID2*	196528	12	46246020	46246020	missense_variant	SNP	T	G	.	15|21	c.4114T>G	p.Leu1372Val	878	0.492
L102tumor	*TSC2*	7249	16	2138304	2138304	missense_variant	SNP	A	C	.	41|42	c.5237A>C	p.His1746Pro	1342	0.7
L102tumor	*AXIN1*	8312	16	396176	396176	stop_gained	SNP	T	A	.	2|11	c.850A>T	p.Arg284Ter	1397	0.679
L102tumor	*TP53*	7157	17	7577547	7577547	missense_variant	SNP	C	A	.	7|11	c.734G>T	p.Gly245Val	1516	0.412
L102tumor	*BAI3*	577	6	70070920	70070920	missense_variant	SNP	A	C	.	29|32	c.3755A>C	p.Gln1252Pro	1836	0.393
L102tumor	*BAI1*	575	8	143545614	143545616	inframe_deletion	DEL	CTG	-	rs777000151	1|30	c.70_72delCTG	p.Leu24del	2511	0.032
L102tumor	*ATRX*	546	X	76907782	76907784	inframe_deletion	DEL	TCC	-	rs398123423	15|35	c.4377_4379delGGA	p.Glu1464del	1175	0.021
L103tumor	*HEG1*	57493	3	124732416	124732417	inframe_insertion	INS	-	GAAGAGGAGGAGGAG	rs754557087	6|17	c.1992_2006dupCTCCTCCTCCTCTTC	p.Ser668_Ser672dup	140	0.043

Sample_ID	Gene_Name	Entrez_Gene_Id	Chromosome	Start_position	End_position	Variant_Classification	Variant_Type	Ref	Alt	dbSNP_RS	Exon_num	cDNA_Change	Protein_Change	Tumor_depth	Tumor_mutant_frequency
L103tumor	*TP53*	7157	17	7579592	7579592	splice_acceptor_variant	SNP	T	A	.	4|11	c.97-2A>T	.	997	0.26
L103tumor	*TERT*	.	5	1295228	1295228	upstream_gene_variant	SNP	G	A	.	N|A	.	.	88	0.125
L104LNM	*ARID2*	196528	12	46243401	46243403	inframe_deletion	DEL	ATT	-	.	14|21	c.1754_1756delATT	p.Asp585_Ser586delinsAla	3936	0.338
L104LNM	*ARID2*	196528	12	46243404	46243404	missense_variant	SNP	C	G	.	14|21	c.1757C>G	p.Ser586Cys	2600	0.339
L104LNM	*ZFHX3*	463	16	72822563	72822564	inframe_insertion	INS	-	TGC	rs376311468	10|10	c.9609_9611dupGCA	p.Gln3204dup	63	0.508
L104LNM	*TP53*	7157	17	7578552	7578552	stop_gained	SNP	G	C	.	5|11	c.378C>G	p.Tyr126Ter	1368	0.606
L104LNM	*TERT*	.	5	1295228	1295228	upstream_gene_variant	SNP	G	A	.	N|A	.	.	239	0.297
L104tumor	*ARID2*	196528	12	46243404	46243404	missense_variant	SNP	C	G	.	14|21	c.1757C>G	p.Ser586Cys	1900	0.075
L104tumor	*ZFHX3*	463	16	72822563	72822564	inframe_insertion	INS	-	TGC	rs376311468	10|10	c.9609_9611dupGCA	p.Gln3204dup	83	0.494
L104tumor	*TP53*	7157	17	7578552	7578552	stop_gained	SNP	G	C	.	5|11	c.378C>G	p.Tyr126Ter	1733	0.1
L104tumor	*TERT*	.	5	1295228	1295228	upstream_gene_variant	SNP	G	A	.	N|A	.	.	208	0.053
L105tumor	-														
L108tumor	*KIT*	3815	4	55592079	55592079	missense_variant	SNP	C	G	rs200518498	9|21	c.1403C>G	p.Pro468Arg	130	0.131
L108tumor	*TP53*	7157	17	7577600	7577611	splice_acceptor_variant	DEL	AGAGCCAACCTA	-	.	7|11	c.673-3_681delTAGGTTGGCTCT	.	440	0.132
L11tumor	*ARID2*	196528	12	46245589	46245589	stop_gained	SNP	C	A	.	15|21	c.3683C>A	p.Ser1228Ter	4366	0.023
L11tumor	*TP53*	7157	17	7578403	7578403	missense_variant	SNP	C	A	.	5|11	c.527G>T	p.Cys176Phe	2507	0.415
L12tumor	*TP53*	7157	17	7577580	7577580	missense_variant	SNP	T	C	rs587780073	7|11	c.701A>G	p.Tyr234Cys	1150	0.746
L15tumor	*ARID1A*	8289	1	27023052	27023052	missense_variant	SNP	C	A	.	1|20	c.158C>A	p.Ala53Asp	31	0.065
L15tumor	*BAI1*	575	8	143592291	143592291	splice_acceptor_variant	SNP	A	C	.	17|30	c.2676-2A>C	.	773	0.031
L15tumor	*BAI1*	575	8	143623763	143623763	missense_variant	SNP	G	C	.	27|30	c.4168G>C	p.Ala1390Pro	1087	0.03

Sample_ID	Gene_Name	Entrez_Gene_Id	Chromosome	Start_position	End_position	Variant_Classification	Variant_Type	Ref	Alt	dbSNP_RS	Exon_num	cDNA_Change	Protein_Change	Tumor_depth	Tumor_mutant_frequency
L15tumor	*CTNNB1*	1499	3	41274859	41274859	missense_variant	SNP	T	G	.	8|15	c.1109T>G	p.Leu370Arg	3859	0.003
L15tumor	*TP53*	7157	17	7577098	7577098	missense_variant	SNP	T	A	.	8|11	c.840A>T	p.Arg280Ser	5206	0.003
L15 re-tumor	*CTNNB1*	1499	3	41274859	41274859	missense_variant	SNP	T	G	.	8|15	c.1109T>G	p.Leu370Arg	5967	0.049
L15 re-tumor	*BAI1*	575	8	143592291	143592291	splice_acceptor_variant	SNP	A	C	.	17|30	c.2676-2A>C	.	283	0.067
L15 re-tumor	*TP53*	7157	17	7577098	7577098	missense_variant	SNP	T	A	.	8|11	c.840A>T	p.Arg280Ser	4245	0.054
L16tumor	*TP53*	7157	17	7577534	7577534	missense_variant	SNP	C	A	rs28934571	7|11	c.747G>T	p.Arg249Ser	1068	0.176
L17tumor	*RB1*	5925	13	48916733	48916733	splice_acceptor_variant	SNP	A	T	.	3|27	c.265-2A>T	.	619	0.16
L17tumor	*RB1*	5925	13	49037866	49037866	splice_acceptor_variant	SNP	G	T	rs587778860	21|27	c.2107-1G>T	.	382	0.118
L17tumor	*TERT*	.	5	1295228	1295228	upstream_gene_variant	SNP	G	A	.	N|A	.	.	94	0.191
L18tumor	*RB1*	5925	13	48934227	48934227	stop_gained	SNP	A		.	7|27	c.682A>T	p.Lys228Ter	2506	0.077
L1tumor	*CTNNB1*	1499	3	41266101	41266101	missense_variant	SNP	C	G	.	3|15	c.98C>G	p.Ser33Cys	2425	0.113
L2tumor	*CTNNB1*	1499	3	41266098	41266098	missense_variant	SNP	A	G	.	3|15	c.95A>G	p.Asp32Gly	1105	0.094
L2tumor	*BAI1*	575	8	143563046	143563046	missense_variant	SNP	T	C	.	10|30	c.2104T>C	p.Tyr702His	380	0.121
L2tumor	*ARID2*	196528	12	46285594	46285594	missense_variant	SNP	C	T	.	17|21	c.4954C>T	p.His1652Tyr	564	0.115
L2tumor	*TP53*	7157	17	7578268	7578268	missense_variant	SNP	A	C	.	6|11	c.581T>G	p.Leu194Arg	468	0.131
L20tumor	*ARID1A*	8289	1	27023883	27023883	missense_variant	SNP	C	T	.	1|20	c.989C>T	p.Ala330Val	478	0.36
L20tumor	*TP53*	7157	17	7578427	7578427	missense_variant	SNP	T	C	.	5|11	c.503A>G	p.His168Arg	802	0.747
L21tumor	-														
L22tumor	*TP53*	7157	17	7577548	7577548	missense_variant	SNP	C	A	.	7|11	c.733G>T	p.Gly245Cys	1355	0.646
L22tumor	*TP53*	7157	17	7577559	7577559	missense_variant	SNP	G	A	rs28934573	7|11	c.722C>T	p.Ser241Phe	1346	0.649
L22tumor	*CDKN2A*	1029	9	21971092	21971092	missense_variant	SNP	C	A	.	2|3	c.266G>T	p.Gly89Val	1425	0.623
L23tumor	*TP53*	7157	17	7579381	7579381	frameshift_variant	DEL	G	-	.	4|11	c.306delC	p.Tyr103ThrfsTer20	2656	0.218

Sample_ID	Gene_Name	Entrez_Gene_Id	Chromosome	Start_position	End_position	Variant_Classification	Variant_Type	Ref	Alt	dbSNP_RS	Exon_num	cDNA_Change	Protein_Change	Tumor_depth	Tumor_mutant_frequency
L23tumor	*CTNNB1*	1499	3	41266101	41266101	missense_variant	SNP	C	T	.	3|15	c.98C>T	p.Ser33Phe	3434	0.093
L24tumor	*TP53*	7157	17	7578542	7578543	frameshift_variant	DEL	GG	-	.	5|11	c.387_388delCC	p.Leu130GlnfsTer18	5032	0.132
L24tumor	*ARID2*	196528	12	46244658	46244658	frameshift_variant	DEL	C	-	.	15|21	c.2753delC	p.Pro918HisfsTer9	6061	0.196
L24tumor	*ARID2*	196528	12	46287503	46287503	stop_gained	SNP	A	T	.	20|21	c.5362A>T	p.Arg1788Ter	1548	0.14
L24tumor	*KIT*	3815	4	55589794	55589794	missense_variant	SNP	C	A	rs758061831	8|21	c.1276C>A	p.Leu426Ile	2759	0.188
L25tumor	*TP53*	7157	17	7578523	7578523	missense_variant	SNP	T	G	.	5|11	c.407A>C	p.Gln136Pro	1047	0.25
L26tumor	*RB1*	5925	13	48881489	48881489	stop_gained	SNP	A	T	.	2|27	c.211A>T	p.Arg71Ter	969	0.498
L26tumor	*RB1*	5925	13	48881499	48881499	missense_variant	SNP	C	G	rs764472420	2|27	c.221C>G	p.Ala74Gly	1037	0.503
L26tumor	*TP53*	7157	17	7572986	7572986	stop_gained	SNP	G	A	.	11|11	c.1123C>T	p.Gln375Ter	1498	0.377
L27tumor	*TP53*	7157	17	7579358	7579358	missense_variant	SNP	C	A	.	4|11	c.329G>T	p.Arg110Leu	1268	0.227
L27tumor	*CTNNB1*	1499	3	41266098	41266098	missense_variant	SNP	A	G	.	3|15	c.95A>G	p.Asp32Gly	2577	0.098
L28tumor	-														
L29tumor	*ZFHX3*	463	16	72822563	72822564	inframe_insertion	INS	-	TGC	rs376311468	10|10	c.9609_9611dupGCA	p.Gln3204dup	25	0.2
L29tumor	*TSC2*	7249	16	2134572	2134572	missense_variant	SNP	C	G	rs45517338	34|42	c.4349C>G	p.Pro1450Arg	2363	0.515
L29tumor	*TP53*	7157	17	7577579	7577579	stop_gained	SNP	G	T	.	7|11	c.702C>A	p.Tyr234Ter	1267	0.37
L29tumor	*CTNNB1*	1499	3	41266097	41266097	missense_variant	SNP	G	C	.	3|15	c.94G>C	p.Asp32His	2178	0.294
L29tumor	*ATRX*	546	X	76938208	76938208	missense_variant	SNP	A	G	rs45624939	9|35	c.2540T>C	p.Phe847Ser	724	0.99
L30tumor	*TSC2*	7249	16	2134598	2134598	stop_gained	SNP	C	T	rs397514959	34|42	c.4375C>T	p.Arg1459Ter	3025	0.03
L30tumor	*TP53*	7157	17	7578524	7578524	missense_variant	SNP	G	C	.	5|11	c.406C>G	p.Gln136Glu	1722	0.157
L31tumor	*TP53*	7157	17	7578457	7578457	missense_variant	SNP	C	A	rs587782144	5|11	c.473G>T	p.Arg158Leu	2484	0.126

Sample_ID	Gene_Name	Entrez_Gene_Id	Chromosome	Start_position	End_position	Variant_Classification	Variant_Type	Ref	Alt	dbSNP_RS	Exon_num	cDNA_Change	Protein_Change	Tumor_depth	Tumor_mutant_frequency
L32tumor	*ARID1A*	8289	1	27092770	27092776	frameshift_variant	DEL	GGACAAG	-	.	9|20	c.2794_2800delCAAGGGA	p.Gln932LeufsTer7	3124	0.218
L32tumor	*ARID1A*	8289	1	27100161	27100161	frameshift_variant	DEL	T	-	.	16|20	c.3958delT	p.Ser1320LeufsTer161	3497	0.268
L32tumor	*CTNNB1*	1499	3	41266098	41266098	missense_variant	SNP	A	G	.	3|15	c.95A>G	p.Asp32Gly	2413	0.298
L32tumor	*TERT*	.	5	1295228	1295228	upstream_gene_variant	SNP	G	A	.	N|A	.	.	137	0.228
L32tumor	*CDKN2A*	1029	9	21971209	21971209	splice_acceptor_variant	SNP	T	A	.	2|3	c.151-2A>T	.	1415	0.3
L33tumor	*TP53*	7157	17	7578211	7578211	missense_variant	SNP	C	G	.	6|11	c.638G>C	p.Arg213Pro	1634	0.362
L33tumor	*TERT*	.	5	1295228	1295228	upstream_gene_variant	SNP	G	A	.	N|A	.	.	284	0.241
L33tumor	*BAI3*	577	6	69653817	69653817	stop_gained	SNP	C	T	.	6|32	c.1126C>T	p.Gln376Ter	2833	0.041
L34tumor	*TERT*	.	5	1295228	1295228	upstream_gene_variant	SNP	G	A	.	N|A	.	.	250	0.412
L34tumor	*MEN1*	4221	11	64577373	64577373	missense_variant	SNP	T	G	.	2|10	c.209A>C	p.Asp70Ala	1923	0.028
L34tumor	*ARID1A*	8289	1	27022940	27022942	inframe_deletion	DEL	CCG	-	rs748085214	1|20	c.60_62delGCC	p.Pro21del	534	0.032
L34tumor	*ARID1A*	8289	1	27100182	27100184	inframe_deletion	DEL	GCA	-	rs374564889	16|20	c.3999_4001delGCA	p.Gln1334del	3127	0.048
L34tumor	*TP53*	7157	17	7577536	7577536	missense_variant	SNP	T	A	rs587782082	7|11	c.745A>T	p.Arg249Trp	1343	0.701
L34tumor	*SETD2*	29072	3	47125632	47125632	stop_gained	SNP	T	A	.	12|21	c.5638A>T	p.Arg1880Ter	2733	0.412
L34tumor	*SETD2*	29072	3	47125834	47125834	missense_variant	SNP	T	A	.	12|21	c.5436A>T	p.Lys1812Asn	1834	0.089
L34tumor	*BAI3*	577	6	69348580	69348580	missense_variant	SNP	C	A	rs759189744	3|32	c.13C>A	p.Arg5Ser	1505	0.461
L34tumor	*BAI1*	575	8	143545614	143545616	inframe_deletion	DEL	CTG	-	rs777000151	1|30	c.70_72delCTG	p.Leu24del	660	0.058
L34tumor	*ATRX*	546	X	76907782	76907784	inframe_deletion	DEL	TCC	-	rs398123423	15|35	c.4377_4379delGGA	p.Glu1464del	1084	0.037
L34tumor	*TP53*	7157	17	7578527	7578527	missense_variant	SNP	A	G	.	5|11	c.403T>C	p.Cys135Arg	2116	0.01

Sample_ID	Gene_Name	Entrez_Gene_Id	Chromosome	Start_position	End_position	Variant_Classification	Variant_Type	Ref	Alt	dbSNP_RS	Exon_num	cDNA_Change	Protein_Change	Tumor_depth	Tumor_mutant_frequency
L35tumor	*ARID1A*	8289	1	27023221	27023222	frameshift_variant	DEL	TA	-	.	1|20	c.327_328delTA	p.Arg110AlafsTer6	238	0.366
L36tumor	*TERT*	.	5	1295228	1295228	upstream_gene_variant	SNP	G	A	.	N|A	.	.	200	0.177
L36tumor	*AXIN1*	8312	16	338187	338187	missense_variant	SNP	C	G	.	11|11	c.2524G>C	p.Glu842Gln	3555	0.03
L38tumor	*TERT*	.	5	1295228	1295228	upstream_gene_variant	SNP	G	A	.	N|A	.	.	247	0.272
L39tumor	*TSC2*	7249	16	2105465	2105465	missense_variant	SNP	A	T	.	6|42	c.544A>T	p.Asn182Tyr	1499	0.087
L39tumor	*TERT*	.	5	1295228	1295228	upstream_gene_variant	SNP	G	A	.	N|A	.	.	285	0.11
L39tumor	*BAI3*	577	6	69653740	69653740	missense_variant	SNP	A	T	.	6|32	c.1049A>T	p.Glu350Val	2885	0.09
L3tumor	-														
L40tumor	*TP53*	7157	17	7579400	7579400	frameshift_variant	DEL	G	-	.	4|11	c.287delC	p.Ser96LeufsTer27	3857	0.364
L40tumor	*TERT*	.	5	1295228	1295228	upstream_gene_variant	SNP	G	A	.	N|A	.	.	339	0.24
L40tumor	*TRIM26*	7726	6	30154123	30154123	stop_gained	SNP	C	A	.	10|10	c.1150G>T	p.Glu384Ter	88	0.193
L40tumor	*TPRN*	286262	9	140087008	140087008	missense_variant	SNP	C	T	rs746612209	2|4	c.1861G>A	p.Glu621Lys	54	0.208
L43tumor	*ARID1A*	8289	1	27100182	27100184	inframe_deletion	DEL	GCA	-	rs374564889	16|20	c.3999_4001delGCA	p.Gln1334del	3710	0.036
L43tumor	*ARID1A*	8289	1	27022940	27022942	inframe_deletion	DEL	CCG	-	rs748085214	1|20	c.60_62delGCC	p.Pro21del	713	0.03
L43tumor	*TSC2*	7249	16	2131788	2131788	missense_variant	SNP	G	A	rs200577441	31|42	c.3803G>A	p.Arg1268His	1324	0.468
L43tumor	*AXIN1*	8312	16	354422	354422	frameshift_variant	DEL	T	-	.	5|11	c.1136delA	p.Lys379ArgfsTer35	4106	0.025
L43tumor	*BAI1*	575	8	143545614	143545616	inframe_deletion	DEL	CTG	-	rs777000151	1|30	c.70_72delCTG	p.Leu24del	788	0.052
L47tumor	*AXIN1*	8312	16	339477	339477	stop_gained	SNP	G	A	.	10|11	c.2425C>T	p.Gln809Ter	702	0.17
L47tumor	*AXIN1*	8312	16	360071	360071	splice_acceptor_variant	SNP	T	A	rs777975737	4|11	c.1020-2A>T	.	521	0.175
L47tumor	*SMARCA4*	6597	19	11152230	11152230	missense_variant	SNP	A	T	.	31|36	c.4418A>T	p.Lys1473Met	496	0.134

Sample_ID	Gene_Name	Entrez_Gene_Id	Chromosome	Start_position	End_position	Variant_Classification	Variant_Type	Ref	Alt	dbSNP_RS	Exon_num	cDNA_Change	Protein_Change	Tumor_depth	Tumor_mutant_frequency
L48tumor	*CDKN2A*	1029	9	21971111	21971111	missense_variant	SNP	G	A	.	2|3	c.247C>T	p.His83Tyr	229	0.279
L48tumor	*ARID2*	196528	12	46285881	46285881	splice_donor_variant	SNP	T	C	.	18|21	c.5147+2T>C	.	208	0.226
L48tumor	*TP53*	7157	17	7578395	7578395	missense_variant	SNP	G	T	rs587780070	5|11	c.535C>A	p.His179Asn	100	0.3
L49T re-tumor	*ARID1A*	8289	1	27106106	27106106	missense_variant	SNP	G	C	rs41303631	20|20	c.5717G>C	p.Arg1906Pro	637	0.495
L49T re-tumor	*TP53*	7157	17	7579592	7579592	splice_acceptor_variant	SNP	T	A	.	4|11	c.97-2A>T	.	460	0.243
L49T re-tumor	*TERT*	.	5	1295228	1295228	upstream_gene_variant	SNP	G	A	.	N|A	.	.	103	0.235
L49tumor	*ARID1A*	8289	1	27106106	27106106	missense_variant	SNP	G	C	rs41303631	20|20	c.5717G>C	p.Arg1906Pro	3888	0.779
L49tumor	*TP53*	7157	17	7579592	7579592	splice_acceptor_variant	SNP	T	A	.	4|11	c.97-2A>T	.	2475	0.568
L49tumor	*TERT*	.	5	1295228	1295228	upstream_gene_variant	SNP	G	A	.	N|A	.	.	952	0.449
L4tumor	*TSC2*	7249	16	2108746	2108746	splice_acceptor_variant	SNP	A	C	rs45517134	10|42	c.849-2A>C	.	469	0.301
L4tumor	*BAI3*	577	6	70048857	70048857	missense_variant	SNP	G	C	.	25|32	c.3238G>C	p.Gly1080Arg	1761	0.182
L50tumor	*AXIN1*	8312	16	354414	354453	splice_acceptor_variant	DEL	GGACCTCCTTCGGCACCCGGTACGTGCGCTGCGAGGGACA	-	.	5|11	c.1117-12_1144delTGTCCCTCGCAGCGCACGTACCGGGTGCCGAAGGAGGTCC	.	5819	0.032

Sample_ID	Gene_Name	Entrez_Gene_Id	Chromosome	Start_position	End_position	Variant_Classification	Variant_Type	Ref	Alt	dbSNP_RS	Exon_num	cDNA_Change	Protein_Change	Tumor_depth	Tumor_mutant_frequency
L52tumor	*TP53*	7157	17	7578475	7578475	missense_variant	SNP	G	A	rs587782705	5|11	c.455C>T	p.Pro152Leu	1828	0.137
L53tumor	*AXIN1*	8312	16	339468	339468	stop_gained	SNP	C	A	.	10|11	c.2434G>T	p.Glu812Ter	958	0.375
L54tumor	*BAI3*	577	6	69348689	69348689	stop_gained	SNP	C	A	rs745590202	3|32	c.122C>A	p.Ser41Ter	2467	0.075
L54tumor	*BAI3*	577	6	69666035	69666035	missense_variant	SNP	G	C	.	7|32	c.1315G>C	p.Gly439Arg	2412	0.079
L54tumor	*BAI3*	577	6	69666035	69666035	missense_variant	SNP	G	C	.	7|32	c.1315G>C	p.Gly439Arg	1549	0.049
L54tumor	*BAI3*	577	6	69348689	69348689	stop_gained	SNP	C	A	rs745590202	3|32	c.122C>A	p.Ser41Ter	1439	0.058
L54tumor	*ST6GALNAC5*	81849	1	77334277	77334282	inframe_deletion	DEL	GCAGCA	-	rs778440501	2|5	c.129_134delGCAGCA	p.Gln48_Gln49del	96	0.042
L55tumor	*RB1*	5925	13	49047527	49047527	splice_donor_variant	SNP	G	T	rs587778850	24|27	c.2520+1G>T	.	327	0.037
L55tumor	*TP53*	7157	17	7577121	7577121	missense_variant	SNP	G	A	.	8|11	c.817C>T	p.Arg273Cys	1887	0.07
L55tumor	*TP53*	7157	17	7577121	7577121	missense_variant	SNP	G	A	.	8|11	c.817C>T	p.Arg273Cys	2839	0.01
L55tumor	*TP53*	7157	17	7577094	7577094	missense_variant	SNP	G	A	.	8|11	c.844C>T	p.Arg282Trp	1348	0.456
L58tumor	-														
L59tumor	*ARID1A*	8289	1	27100182	27100184	inframe_deletion	DEL	GCA	-	rs374564889	16|20	c.3999_4001delGCA	p.Gln1334del	1858	0.026
L59tumor	*ARID1A*	8289	1	27023451	27023453	inframe_deletion	DEL	GCG	-	rs772367471	1|20	c.567_569delCGG	p.Gly191del	1370	0.02
L59tumor	*CTNNB1*	1499	3	41266136	41266136	missense_variant	SNP	T	C	rs121913407	3|15	c.133T>C	p.Ser45Pro	998	0.501
L59tumor	*BAI1*	575	8	143545614	143545616	inframe_deletion	DEL	CTG	-	rs777000151	1|30	c.70_72delCTG	p.Leu24del	1376	0.052
L59tumor	*ARID1A*	8289	1	27022940	27022942	inframe_deletion	DEL	CCG	-	rs748085214	1|20	c.60_62delGCC	p.Pro21del	376	0.021
L60tumor	*TP53*	7157	17	7578188	7578188	stop_gained	SNP	C	A	.	6|11	c.661G>T	p.Glu221Ter	1982	0.503
L61tumor	-														

Sample_ID	Gene_Name	Entrez_Gene_Id	Chromosome	Start_position	End_position	Variant_Classification	Variant_Type	Ref	Alt	dbSNP_RS	Exon_num	cDNA_Change	Protein_Change	Tumor_depth	Tumor_mutant_frequency
L62Ttumor	*ARID1A*	8289	1	27023601	27023602	frameshift_variant	INS	-	TGGCACT	.	1|20	c.708_714dupTGGCACT	p.Pro239TrpfsTer163	2141	0.331
L62Ttumor	*ARID1A*	8289	1	27107135	27107136	frameshift_variant	INS	-	A	.	20|20	c.6747dupA	p.Glu2250ArgfsTer28	4146	0.368
L62Ttumor	*ZFHX3*	463	16	72822563	72822564	inframe_insertion	INS	-	TGC	rs376311468	10|10	c.9609_9611dupGCA	p.Gln3204dup	45	0.244
L62Ttumor	*SMARCA4*	6597	19	11143973	11143973	missense_variant	SNP	A	T	.	27|36	c.3554A>T	p.Gln1185Leu	1060	0.253
L62Ttumor	*CTNNB1*	1499	3	41266104	41266104	missense_variant	SNP	G	T	rs28931589	3|15	c.101G>T	p.Gly34Val	2790	0.361
L62Ttumor	*TERT*	.	5	1295228	1295228	upstream_gene_variant	SNP	G	A	.	N|A	.	.	313	0.26
L63tumor	*TERT*	.	5	1295228	1295228	upstream_gene_variant	SNP	G	A	.	N|A	.	.	162	0.21
L63tumor	*ARID1A*	8289	1	27022940	27022942	inframe_deletion	DEL	CCG	-	rs748085214	1|20	c.60_62delGCC	p.Pro21del	413	0.027
L63tumor	*ARID1A*	8289	1	27100182	27100184	inframe_deletion	DEL	GCA	-	rs374564889	16|20	c.3999_4001delGCA	p.Gln1334del	2273	0.038
L63tumor	*TP53*	7157	17	7578206	7578206	missense_variant	SNP	T	C	.	6|11	c.643A>G	p.Ser215Gly	932	0.242
L63tumor	*SMARCA4*	6597	19	11145716	11145718	inframe_deletion	DEL	GAG	-	rs148514235	30|36	c.4090_4092delGAG	p.Glu1364del	1772	0.02
L63tumor	*SETD2*	29072	3	47164775	47164775	missense_variant	SNP	T	C	.	3|21	c.1351A>G	p.Thr451Ala	1427	0.079
L63tumor	*BAI1*	575	8	143545614	143545616	inframe_deletion	DEL	CTG	-	rs777000151	1|30	c.70_72delCTG	p.Leu24del	362	0.061
L64tumor	*ARID1A*	8289	1	27101382	27101382	missense_variant	SNP	A	C	.	18|20	c.4664A>C	p.Tyr1555Ser	1518	0.02
L64tumor	*TP53*	7157	17	7579432	7579432	frameshift_variant	DEL	A	-	rs775515332	4|11	c.255delT	p.Ala86HisfsTer37	1010	0.025
L69tumor	*TP53*	7157	17	7578263	7578263	missense_variant	SNP	G	C	.	6|11	c.586C>G	p.Arg196Gly	2411	0.22

Sample_ID	Gene_Name	Entrez_Gene_Id	Chromosome	Start_position	End_position	Variant_Classification	Variant_Type	Ref	Alt	dbSNP_RS	Exon_num	cDNA_Change	Protein_Change	Tumor_depth	Tumor_mutant_frequency
L69tumor	*ARID1A*	8289	1	27023853	27023877	frameshift_variant	DEL	CCCAGGACGGGGGCGCCGGCAAGGG	-	.	1|20	c.962_986delAGGACGGGGGCGCCGGCAAGGGCCC	p.Gln321ArgfsTer34	5950	0.104
L69tumor	*CTNNB1*	1499	3	41266113	41266113	missense_variant	SNP	C	T	.	3|15	c.110C>T	p.Ser37Phe	2845	0.183
L69tumor	*KIT*	3815	4	55561756	55561756	missense_variant	SNP	G	A	rs376469897	2|21	c.146G>A	p.Arg49His	1973	0.209
L69tumor	*TERT*	.	5	1295228	1295228	upstream_gene_variant	SNP	G	A	.	N|A	.	.	400	0.236
L6tumor	-														
L70tumor	*MEN1*	4221	11	64577373	64577373	missense_variant	SNP	T	G	.	2|10	c.209A>C	p.Asp70Ala	671	0.037
L70tumor	*ARID2*	196528	12	46246488	46246488	missense_variant	SNP	G	T	rs761166036	15|21	c.4582G>T	p.Ala1528Ser	1088	0.519
L70tumor	*ARID1A*	8289	1	27022940	27022942	inframe_deletion	DEL	CCG	-	rs748085214	1|20	c.60_62delGCC	p.Pro21del	232	0.056
L70tumor	*ARID1A*	8289	1	27100182	27100184	inframe_deletion	DEL	GCA	-	rs374564889	16|20	c.3999_4001delGCA	p.Gln1334del	1080	0.045
L70tumor	*BAI1*	575	8	143545614	143545616	inframe_deletion	DEL	CTG	-	rs777000151	1|30	c.70_72delCTG	p.Leu24del	306	0.056
L72tumor	*AXIN1*	8312	16	396361	396361	missense_variant	SNP	C	T	rs763104413	2|11	c.665G>A	p.Ser222Asn	895	0.635
L72tumor	*TP53*	7157	17	7577534	7577534	missense_variant	SNP	C	A	rs28934571	7|11	c.747G>T	p.Arg249Ser	837	0.278
L73tumor	-														
L74tumor	-														
L75tumor	-														

Sample_ID	Gene_Name	Entrez_Gene_Id	Chromosome	Start_position	End_position	Variant_Classification	Variant_Type	Ref	Alt	dbSNP_RS	Exon_num	cDNA_Change	Protein_Change	Tumor_depth	Tumor_mutant_frequency
L76tumor	*SETD2*	29072	3	47125476	47125476	stop_gained	SNP	G	A	.	12|21	c.5794C>T	p.Gln1932Ter	698	0.099
L76tumor	*SETD2*	29072	3	47164730	47164730	stop_gained	SNP	T	A	.	3|21	c.1396A>T	p.Lys466Ter	652	0.112
L76tumor	*SETD2*	29072	3	47164731	47164731	stop_gained	SNP	A	T	.	3|21	c.1395T>A	p.Tyr465Ter	667	0.112
L76tumor	*TSC2*	7249	16	2114340	2114340	missense_variant	SNP	T	A	.	15|42	c.1511T>A	p.Val504Asp	1079	0.038
L76tumor	*TP53*	7157	17	7577610	7577610	splice_acceptor_variant	SNP	T	A	.	7|11	c.673-2A>T	.	787	0.121
L76tumor	*TP53*	7157	17	7578550	7578550	missense_variant	SNP	G	C	rs730881999	5|11	c.380C>G	p.Ser127Cys	753	0.143
L77tumor	-														
L78tumor	-														
L79tumor	-														
L7tumor	*TP53*	7157	17	7576927	7576927	splice_acceptor_variant	SNP	C	T	rs587781702	9|11	c.920-1G>A	.	1285	0.639
L7tumor	*TERT*	.	5	1295228	1295228	upstream_gene_variant	SNP	G	A	.	N|A	.	.	374	0.407
L81tumor	*ARID1A*	8289	1	27100182	27100184	inframe_deletion	DEL	GCA	-	rs374564889	16|20	c.3999_4001delGCA	p.Gln1334del	2863	0.03
L81tumor	*ARID1A*	8289	1	27022940	27022942	inframe_deletion	DEL	CCG	-	rs748085214	1|20	c.60_62delGCC	p.Pro21del	2540	0.038
L81tumor	*BAI3*	577	6	69944975	69944975	missense_variant	SNP	C	G	.	19|32	c.2659C>G	p.Leu887Val	1371	0.056
L81tumor	*BAI1*	575	8	143545614	143545616	inframe_deletion	DEL	CTG	-	rs777000151	1|30	c.70_72delCTG	p.Leu24del	3185	0.057
L82tumor	*BAI3*	577	6	70098643	70098643	missense_variant	SNP	A	T	.	32|32	c.4429A>T	p.Ser1477Cys	804	0.057
L83tumor	*CTNNB1*	1499	3	41266124	41266124	missense_variant	SNP	A	G	.	3|15	c.121A>G	p.Thr41Ala	1906	0.091
L83tumor	*TERT*	.	5	1295228	1295228	upstream_gene_variant	SNP	G	A	.	N|A	.	.	253	0.116
L84tumor	*TP53*	7157	17	7578188	7578188	stop_gained	SNP	C	A	.	6|11	c.661G>T	p.Glu221Ter	902	0.215
L85tumor	*TP53*	7157	17	7577082	7577082	missense_variant	SNP	C	T	.	8|11	c.856G>A	p.Glu286Lys	2442	0.209
L86tumor	*TERT*	.	5	1295228	1295228	upstream_gene_variant	SNP	G	A	.	N|A	.	.	277	0.116
L89tumor	-														

Sample_ID	Gene_Name	Entrez_Gene_Id	Chromosome	Start_position	End_position	Variant_Classification	Variant_Type	Ref	Alt	dbSNP_RS	Exon_num	cDNA_Change	Protein_Change	Tumor_depth	Tumor_mutant_frequency
L8tumor	*AXIN1*	8312	16	347983	347983	frameshift_variant	DEL	C	-	rs760961378	6|11	c.1523delG	p.Gly508ValfsTer197	4476	0.01
L92tumor	*TP53*	7157	17	7576870	7576870	stop_gained	SNP	C	A	.	9|11	c.976G>T	p.Glu326Ter	832	0.587
L92tumor	*CTNNB1*	1499	3	41266113	41266113	missense_variant	SNP	C	T	.	3|15	c.110C>T	p.Ser37Phe	1884	0.228
L92tumor	*TERT*	.	5	1295228	1295228	upstream_gene_variant	SNP	G	A	.	N|A	.	.	313	0.497
L93tumor	-														
L95tumor	-														
L96tumor	*CTNNB1*	1499	3	41266098	41266098	missense_variant	SNP	A	G	.	3|15	c.95A>G	p.Asp32Gly	819	0.275
L96tumor	*TP53*	7157	17	7578406	7578406	missense_variant	SNP	C	T	.	5|11	c.524G>A	p.Arg175His	715	0.523
L97tumor	-														
L98tumor	-														
L99tumor	*RB1*	5925	13	48919223	48919223	stop_gained	SNP	A	T	.	4|27	c.388A>T	p.Lys130Ter	37	0.297
L99tumor	*TP53*	7157	17	7578469	7578469	missense_variant	SNP	C	T	rs762846821	5|11	c.461G>A	p.Gly154Asp	522	0.264
L99tumor	*TP53*	7157	17	7578444	7578467	inframe_deletion	DEL	GATGGCCATGGCGCGGACGCGGGT	-	.	5|11	c.463_486delACCCGCGTCCGCGCCATGGCCATC	p.Thr155_Ile162del	1067	0.191
L99tumor	*TP53*	7157	17	7574035	7574035	splice_acceptor_variant	SNP	T	A	.	10|11	c.994-2A>T	.	439	0.146
L9tumor	-														

Table S4. Tumor mutations detected in corresponding CTCs

	Gene	Chromosome	Start_position	End_position	Ref	Alt	dbSNP_RS	Exon_num	CTC site	Consistency with tumor	Positive ratio(%)
L1	*CTNNB1*	3	41266101	41266101	C	G	.	3|15	CG	YES	14.86697966
L2	*CTNNB1*	3	41266098	41266098	A	G	.	3|15	AG	YES	8.016032064
L2	*BAI1*	8	143563046	143563046	T	C	.	10|30	CT	YES	13.76811594
L2	*ARID2*	12	46285594	46285594	C	T	.	17|21	CT	YES	15.30864198
L2	*TP53*	17	7578268	7578268	A	C	.	6|11	AC	YES	19.01041667
L4	*TSC2*	16	2108746	2108746	A	C	rs45517134	10|42	AC	YES	30.45454545
L4	*BAI3*	6	70048857	70048857	G	C	.	25|32	GC	YES	12.92035398
L7	*TP53*	17	7576927	7576927	C	T	rs587781702	9|11	CT	YES	32.33944954
L7	*TERT*	5	1295228	1295228	G	A	.	N|A	GA	YES	43.47826087
L8	*AXIN1*	16	347983	347983	C	-	rs760961378	6|11	CC	NO	0
L11	*ARID2*	12	46245589	46245589	C	A	.	15|21	CC	NO	0
L11	*TP53*	17	7578403	7578403	C	A	.	5|11	CA	YES	53.53016688
L12	*TP53*	17	7577580	7577580	T	C	rs587780073	7|11	CT	YES	82.73244782
L15	*ARID1A*	1	27023052	27023052	C	A	.	1|20	CC	NO	0
L15	*BAI1*	8	143592291	143592291	A	C	.	17|30	*NA*	*NA*	*NA*
L15	*BAI1*	8	143623763	143623763	G	C	.	27|30	*NA*	*NA*	*NA*
L15	*CTNNB1*	3	41274859	41274859	T	G	.	8|15	TT	NO	0
L15	*TP53*	17	7577098	7577098	T	A	.	8|11	TT	NO	0
L16	*TP53*	17	7577534	7577534	C	A	rs28934571	7|11	CA	YES	11.51385928
L17	*RB1*	13	48916733	48916733	A	T	.	3|27	AA	NO	0
L17	*RB1*	13	49037866	49037866	G	T	rs587778860	21|27	GT	YES	16.23931624
L17	*TERT*	5	1295228	1295228	G	A	.	N|A	*NA*	*NA*	*NA*
L18	*RB1*	13	48934227	48934227	A	T	.	7|27	AA	NO	0
L20	*ARID1A*	1	27023883	27023883	C	T	.	1|20	CT	YES	54.84693878
L20	*TP53*	17	7578427	7578427	T	C	.	5|11	TC	YES	75.64766839
L22	*TP53*	17	7577548	7577548	C	A	.	7|11	CA	YES	9.108910891
L22	*TP53*	17	7577559	7577559	G	A	rs28934573	7|11	GA	YES	67.83088235
L22	*CDKN2A*	9	21971092	21971092	C	A	.	2|3	CA	YES	17.74647887

	Gene	Chromosome	Start_position	End_position	Ref	Alt	dbSNP_RS	Exon_num	CTC site	Consistency with tumor	Positive ratio(%)
L23	*TP53*	17	7579381	7579381	G	-	.	4|11	G/-	YES	50
L23	*CTNNB1*	3	41266101	41266101	C	T	.	3|15	CT	YES	7.105719237
L24	*TP53*	17	7578542	7578543	GG	-	.	5|11	GG/-	YES	50
L24	*ARID2*	12	46244658	46244658	C	-	.	15|21	C/-	YES	50
L24	*ARID2*	12	46287503	46287503	A	T	.	20|21	AT	YES	84.32539683
L24	*KIT*	4	55589794	55589794	C	A	rs758061831	8|21	CC	NO	0
L25	*TP53*	17	7578523	7578523	T	G	.	5|11	TG	YES	22.5950783
L27	*TP53*	17	7579358	7579358	C	A	.	4|11	CA	YES	19.88304094
L27	*CTNNB1*	3	41266098	41266098	A	G	.	3|15	AG	YES	11.15618661
L29	*CTNNB1*	3	41266097	41266097	G	A	.	3|15	GA	YES	17.5313059
L29	*ATRX*	X	76938208	76938208	A	G	rs45624939	9|35	AG	YES	5.722070845
L29	*ZFHX3*	16	72822563	72822564	-	TGC	rs376311468	10|10	TGC/-	YES	50
L29	*TSC2*	16	2134572	2134572	C	G	rs45517338	34|42	CC	NO	0
L29	*TP53*	17	7577579	7577579	G	T	.	7|11	*NA*	*NA*	*NA*
L30	*TSC2*	16	2134598	2134598	C	T	rs397514959	34|42	CC	NO	0
L30	*TP53*	17	7578524	7578524	G	C	.	5|11	GC	YES	16
L31	*TP53*	17	7578457	7578457	C	A	rs587782144	5|11	CA	YES	19.0376569
L32	*CTNNB1*	3	41266098	41266098	A	G	.	3|15	AG	YES	24.50805009
L32	*TERT*	5	1295228	1295228	G	A	.	N|A	GA	YES	32.98755187
L32	*ARID1A*	1	27092770	27092776	GGA(	-	.	9|20	GACAAC	YES	50
L32	*ARID1A*	1	27100161	27100161	T	-	.	16|20	T/-	YES	50
L32	*CDKN2A*	9	21971209	21971209	T	A	.	2|3	TA	YES	35.79881657
L33	*TP53*	17	7578211	7578211	C	G	.	6|11	CG	YES	34.19811321
L33	*TERT*	5	1295228	1295228	G	A	.	N|A	GA	YES	34.34343434
L33	*BAI3*	6	69653817	69653817	C	T	.	6|32	CC	NO	0
L34	*TERT*	5	1295228	1295228	G	A	.	N|A	GA	YES	43.06418219
L34	*MEN1*	11	64577373	64577373	T	G	.	2|10	TT	NO	0
L34	*ARID1A*	1	27022940	27022942	CCG	-	rs748085214	1|20	CCG/CCC	NO	0
L34	*ARID1A*	1	27100182	27100184	GCA	-	rs374564889	16|20	GCA	NO	0

	Gene	Chromosome	Start_position	End_position	Ref	Alt	dbSNP_RS	Exon_num	CTC site	Consistency with tumor	Positive ratio(%)
L34	*TP53*	17	7577536	7577536	T	A	rs587782082	7|11	TA	YES	64.1509434
L34	*SETD2*	3	47125632	47125632	T	A	.	12|21	AT	YES	35.52036199
L34	*SETD2*	3	47125834	47125834	T	A	.	12|21	AT	YES	8.586762075
L34	*BAI3*	6	69348580	69348580	C	A	rs759189744	3|32	AC	YES	15.19507187
L34	*BAI1*	8	143545614	143545616	CTG	-	rs777000151	1|30	CTG/CTG	NO	0
L34	*ATRX*	X	76907782	76907784	TCC	-	rs398123423	15|35	TCC/TCC	NO	0
L34	*TP53*	17	7578527	7578527	A	G	.	5|11	AA	NO	0
L35	*ARID1A*	1	27023221	27023222	TA	-	.	1|20	TA/-	YES	50
L36	*TERT*	5	1295228	1295228	G	A	.	N|A	GA	YES	23.42857143
L36	*AXIN1*	16	338187	338187	C	G	.	11|11	CC	NO	0
L38	*TERT*	5	1295228	1295228	G	A	.	N|A	GA	YES	30.75313808
L39	*TSC2*	16	2105465	2105465	A	T	.	6|42	AA	NO	0
L39	*TERT*	5	1295228	1295228	G	A	.	N|A	GA	YES	18.00766284
L39	*BAI3*	6	69653740	69653740	A	T	.	6|32	AT	YES	7.992202729
L43	*ARID1A*	1	27100182	27100184	GCA	-	rs374564889	16|20	GCA/GCA	NO	0
L43	*ARID1A*	1	27022940	27022942	CCG	-	rs748085214	1|20	CCG/CCG	NO	0
L43	*TSC2*	16	2131788	2131788	G	A	rs200577441	31|42	GA	YES	59.45273632
L43	*AXIN1*	16	354422	354422	T	-	.	5|11	TT	NO	0
L43	*BAI1*	8	143545614	143545616	CTG	-	rs777000151	1|30	*NA*	*NA*	*NA*
L48	*CDKN2A*	9	21971111	21971111	G	A	AG	2|3	GA	YES	17.30769231
L48	*ARID2*	12	46285881	46285881	T	C	.	18|21	CT	YES	32.8011611
L48	*TP53*	17	7578395	7578395	G	T	rs587780070	5|11	TG	YES	33.61227336
L49	*ARID1A*	1	27106106	27106106	G	C	rs41303631	20|20	GC	YES	19.8938992
L49	*TP53*	17	7579592	7579592	T	A	.	4|11	TA	YES	37.11538462
L49	*TERT*	5	1295228	1295228	G	A	.	N|A	GA	YES	46.62813102
L52	*TP53*	17	7578475	7578475	G	A	rs587782705	5|11	GA	YES	21.2371134
L53	*AXIN1*	16	339468	339468	C	A	.	10|11	CA	YES	43.80530973
L54	*BAI3*	6	69666035	69666035	G	C	.	7|32	GC	YES	14.44141689
L54	*BAI3*	6	69348689	69348689	C	A	rs745590202	3|32	AC	YES	10

	Gene	Chromosome	Start_position	End_position	Ref	Alt	dbSNP_RS	Exon_num	CTC site	Consistency with tumor	Positive ratio(%)
L54	*ST6GALNA(*	1	77334277	77334282	GCA(	-	rs778440501	2|5	*NA*	*NA*	*NA*
L55	*RB1*	13	49047527	49047527	G	T	rs587778850	24|27	GG	NO	0
L55	*TP53*	17	7577121	7577121	G	A	.	8|11	GG	NO	0
L55	*TP53*	17	7577094	7577094	G	A	.	8|11	GG	NO	0
L59	*CTNNB1*	3	41266136	41266136	T	C	rs121913407	3|15	CT	YES	47.2361809
L59	*ARID1A*	1	27100182	27100184	GCA	-	rs374564889	16|20	*NA*	*NA*	*NA*
L59	*ARID1A*	1	27023451	27023453	GCG	-	rs772367471	1|20	GCG/GCC	NO	0
L59	*BAI1*	8	143545614	143545616	CTG	-	rs777000151	1|30	CTG/CTG	NO	0
L59	*ARID1A*	1	27022940	27022942	CCG	-	rs748085214	1|20	CCG/CCC	NO	0
L60	*TP53*	17	7578188	7578188	C	A	.	6|11	CA	YES	46.84210526
L62	*ARID1A*	1	27023601	27023602	-	TGGC	.	1|20	GGCACT	YES	50
L62	*ARID1A*	1	27107135	27107136	-	A	.	20|20	A/-	YES	50
L62	*ZFHX3*	16	72822563	72822564	-	TGC	rs376311468	10|10	TGC/-	YES	50
L62	*SMARCA4*	19	11143973	11143973	A	T	.	27|36	AT	YES	34.7133758
L62	*CTNNB1*	3	41266104	41266104	G	T	rs28931589	3|15	GT	YES	30.65539112
L62	*TERT*	5	1295228	1295228	G	A	.	N|A	GA	YES	27.5142315
L63	*TERT*	5	1295228	1295228	G	A	.	N|A	GA	YES	22.60869565
L63	*ARID1A*	1	27022940	27022942	CCG	-	rs748085214	1|20	CCG/CCC	NO	0
L63	*ARID1A*	1	27100182	27100184	GCA	-	rs374564889	16|20	GCA/GCA	NO	0
L63	*TP53*	17	7578206	7578206	T	C	.	6|11	TC	YES	17.94258373
L63	*SMARCA4*	19	11145716	11145718	GAG	-	rs148514235	30|36	GAG/GAC	NO	0
L63	*SETD2*	3	47164775	47164775	T	C	.	3|21	TT	NO	0
L63	*BAI1*	8	143545614	143545616	CTG	-	rs777000151	1|30	CTG/CTG	NO	0
L64	*TP53*	17	7579432	7579432	A	-	rs775515332	4|11	AA	NO	0
L64	*ARID1A*	1	27101382	27101382	A	C	.	18|20	AA	NO	0
L64	*RB1*	13	48878061	48878061	A	C	.	1|27	AA	NO	0
L69	*TP53*	17	7578263	7578263	G	C	.	6|11	GC	YES	30.20527859
L69	*ARID1A*	1	27023853	27023877	CCCA	-	.	1|20	*NA*	*NA*	*NA*
L69	*CTNNB1*	3	41266113	41266113	C	T	.	3|15	CT	YES	20.07104796

	Gene	Chromosome	Start_position	End_position	Ref	Alt	dbSNP_RS	Exon_num	CTC site	Consistency with tumor	Positive ratio(%)
L69	*KIT*	4	55561756	55561756	G	A	rs376469897	2|21	AG	YES	19.28020566
L69	*TERT*	5	1295228	1295228	G	A	.	N|A	GA	YES	29.01960784
L70	*MEN1*	11	64577373	64577373	T	G	.	2|10	TT	NO	0
L70	*ARID2*	12	46246488	46246488	G	T	rs761166036	15|21	TG	YES	63.10679612
L70	*ARID1A*	1	27022940	27022942	CCG	-	rs748085214	1|20	*NA*	*NA*	*NA*
L70	*ARID1A*	1	27100182	27100184	GCA	-	rs374564889	16|20	GCA/GCA	NO	0
L70	*BAI1*	8	143545614	143545616	CTG	-	rs777000151	1|30	CTG/CTG	NO	0
L72	*RB1*	13	49030485	49030485	G	C	rs483352690	19|27	GG	NO	0
L72	*AXIN1*	16	396361	396361	C	T	rs763104413	2|11	CT	YES	63.53211009
L72	*TP53*	17	7577534	7577534	C	A	rs28934571	7|11	*NA*	*NA*	*NA*
L76	*SETD2*	3	47125476	47125476	G	A	.	12|21	GG	NO	0
L76	*SETD2*	3	47164730	47164730	T	A	.	3|21	TT	NO	0
L76	*SETD2*	3	47164731	47164731	A	T	.	3|21	AA	NO	0
L76	*TSC2*	16	2114340	2114340	T	A	.	15|42	*NA*	*NA*	*NA*
L76	*TP53*	17	7577610	7577610	T	A	.	7|11	TT	NO	0
L76	*TP53*	17	7578550	7578550	G	C	rs730881999	5|11	GG	NO	0
L81	*ARID1A*	1	27100182	27100184	GCA	-	rs374564889	16|20	GCA	NO	0
L81	*ARID1A*	1	27022940	27022942	CCG	-	rs748085214	1|20	*NA*	*NA*	*NA*
L81	*BAI3*	6	69944975	69944975	C	G	.	19|32	CG	YES	7.226107226
L81	*BAI1*	8	143545614	143545616	CTG	-	rs777000151	1|30	CTG/CTG	NO	0
L82	*BAI3*	6	70098643	70098643	A	T	.	32|32	AT	YES	11.25541126
L83	*CTNNB1*	3	41266124	41266124	A	G	.	3|15	GA	YES	14.50094162
L83	*TERT*	5	1295228	1295228	G	A	.	N|A	GA	YES	20.63492063
L84	*TP53*	17	7578188	7578188	C	A	.	6|11	CG	YES	21.74757282
L85	*TP53*	17	7577082	7577082	C	T	.	8|11	CT	YES	20.66549912
L86	*TERT*	5	1295228	1295228	G	A	.	N|A	GA	YES	16.42105263
L92	*TP53*	17	7576870	7576870	C	A	.	9|11	CA	YES	19.01709402
L92	*CTNNB1*	3	41266113	41266113	C	T	.	3|15	CT	YES	27.7486911
L92	*TERT*	5	1295228	1295228	G	A	.	N|A	GA	YES	12.44725738

	Gene	Chromosome	Start_position	End_position	Ref	Alt	dbSNP_RS	Exon_num	CTC site	Consistency with tumor	Positive ratio(%)
L96	*CTNNB1*	3	41266098	41266098	A	G	.	3|15	AG	YES	14.34782609
L96	*TP53*	17	7578406	7578406	C	T	.	5|11	CT	YES	39.41176471
L99	*RB1*	13	48919223	48919223	A	T	.	4|27	AA	NO	0
L99	*TP53*	17	7578469	7578469	C	T	rs762846821	5|11	CC	NO	0
L99	*TP53*	17	7578444	7578467	GATC	-	.	5|11	GGT/GAT	NO	0
L99	*TP53*	17	7574035	7574035	T	A	.	10|11	TA	YES	14.28571429
L100	*AXIN1*	16	396792	396792	G	-	.	2|11	G/-	YES	50
L100	*ARID2*	12	46245966	46245966	A	T	.	15|21	AT	YES	12.19512195
L100	*TSC2*	16	2108755	2108755	A	G	rs1800748	10|42	AG	YES	43.7007874
L100	*TSC2*	16	2112569	2112569	G	T	.	13|42	GT	YES	29.92957746
L100	*TSC2*	16	2121870	2121870	G	A	rs200494044	19|42	AG	YES	9.554140127
L100	*AXIN1*	16	396815	396815	C	A	.	2|11	CA	YES	30.73047859
L100	*PIK3R5*	17	8792116	8792118	CCT	-	rs757971428	10|19	CCT/CCT	NO	0
L102	*TP53*	17	7577548	7577548	C	T	.	7|11	CT	YES	42.48496994
L102	*ARID1A*	1	27100182	27100184	GCA	-	rs374564889	16|20	GCA/GCA	NO	0
L102	*ARID2*	12	46246020	46246020	T	G	.	15|21	TG	YES	62.94027565
L102	*TSC2*	16	2138304	2138304	A	C	.	41|42	AC	YES	22.53164557
L102	*AXIN1*	16	396176	396176	T	A	.	2|11	TA	YES	35.14492754
L102	*TP53*	17	7577547	7577547	C	A	.	7|11	CA	YES	47.86641929
L102	*BAI3*	6	70070920	70070920	A	C	.	29|32	AC	YES	47.5
L102	*BAI1*	8	143545614	143545616	CTG	-	rs777000151	1|30	CTG/CTG	NO	0
L102	*ATRX*	X	76907782	76907784	TCC	-	rs398123423	15|35	TCC/TCC	NO	0
L103	*HEG1*	3	124732416	124732417	-	GAAC	rs754557087	6|17	GGAGGA	YES	50
L104	*ARID2*	12	46243401	46243403	ATT	-	.	14|21	ATT/-	YES	50
L104	*ARID2*	12	46243404	46243404	C	G	.	14|21	GC	YES	21.48760331
L104	*ZFHX3*	16	72822563	72822564	-	TGC	rs376311468	10|10	TGC/-	YES	50
L104	*TP53*	17	7578552	7578552	G	C	.	5|11	CG	YES	20.83333333
L104	*TERT*	5	1295228	1295228	G	A	.	N|A	AG	YES	27.31376975
L108	*KIT*	4	55592079	55592079	C	G	rs200518498	9|21	CG	YES	18.16143498

	Gene	Chromosome	Start_position	End_position	Ref	Alt	dbSNP_RS	Exon_num	CTC site	Consistency with tumor	Positive ratio(%)
L108	*TP53*	17	7577600	7577611	AGA(	-	.	7|11	*NA*	*NA*	*NA*

Table S5. Correlation between clinical parameters and CTC number or proliferative CTC percentage in peripheral blood

	N	CTC Nmuber (per/7.5ml) Mean ± SD (range)	*P* value	PCP (%) Mean ± SD (range)	*P* value
Age (years)			0.476		0.0766
⩽60	193	16 ± 16.45 (0-140)		22.30 ± 23.52 (0-92.88)	
>60	77	14 ± 14.83 (0-75)		16.67 ± 23.65 (0-84.62)	
Sex			0.7685		0.2162
Female	42	15.5 ± 12.20 (0-60)		17.50 ± 21.45 (0-76.08)	
Male	228	15 ± 16.59 (0-140)		20.83 ± 23.94 (0-92.88)	
AFP			0.0041		0.0135
⩽20	123	13 ± 18.04 (0-140)		16.67 ± 23.87 (0-92.88)	
>20 ng/ml	147	17 ± 13.95 (0-75)		25.00 ± 23.17 (0-90.00)	
Prior treatment			0.0033		0.247
Absent	187	15 ± 15.14 (0-140)		20.00 ± 23.45 (0-92.88)	
Present	83	18 ± 17.25 (0-106)		25.00 ± 23.94 (0-87.30)	
Surgical procedure			0.0063		0.0481
Hepatectomy	139	14 ± 14.26 (0-75)		18.52 ± 24.71 (0-92.88)	
Liver transplantation	131	17 ± 17.47 (0-140)		26.67 ± 22.28 (0-86.16)	
Differentiation			0.8746		0.8008
Good	13	13 ± 14.68 (0-45)		18.89 ± 26.68 (0-72.22)	
Moderate	171	15 ± 15.52 (0-140)		20.83 ± 23.32 (0-92.88)	
Poor	84	15 ± 16.68 (0-106)		18.90 ± 23.81 (0-91.67)	
MVI			<0.0001		<0.0001
Absent	193	13 ± 11.58 (0-106)		17.22 ± 20.16 (0-92.88)	
Present	77	23 ± 20.26 (0-140)		40.00 ± 26.79 (0-91.67)	
Tumor thrombosis			<0.0001		<0.0001
Absent	228	14 ± 11.26 (0-60)		17.48 ± 21.23 (0-92.88)	
Present	42	25 ± 26.19 (10-140)		47.41 ± 25.71 (0-86.16)	
Largest tumor diameter (mm)			0.0003		0.0002
≤50	185	14 ± 10.93 (0-60.22)		17.53 ± 21.81 (0-92.88)	
>50	85	17 ± 22.26 (0-140)		35.00 ± 25.34 (0-91.67)	
Tumor number			<0.0001		0.0017
Single	225	15 ± 11.75 (0-71)		19.35 ± 22.64 (0-92.88)	

	N	CTC Nmuber (per/7.5ml)<br>Mean ± SD (range)	*P* value	PCP (%)<br>Mean ± SD (range)	*P* value
Multi	45	23 ± 25.56 (5-140)		38.89 ± 25.60 (0-86.16)	
AJCC stage			<0.0001		0.0005
1	153	12 ± 8.28 (0-63)		17.34 ± 22.01 (0-92.88)	
2	93	19.67 ± 15.13 (0-106)		24.72 ± 24.82 (0-91.67)	
3+4	24	35 ± 25.14 (11-140)		38.89 ± 22.23 (0-87.30)	
BCLC stage			<0.0001		<0.0001
A	207	13 ± 9.38 (0-63)		18.21 ± 22.00 (0-92.88)	
B	34	23 ± 17.12 (3-106)		25.04 ± 22.26 (0-91.67)	
C	29	38.67 ± 24.62 (17-140)		44.44 ± 27.39 (0-87.30)	

AJCC, American Joint Committee on Cancer; BCLC, Barcelona clinic liver cancer stage; MVI, microvascular invasion; AFP, alpha-fetoprotein; CTC, circulating tumor cell; PCP, proliferative CTC percentage.

Table S6. Cox regression model to evaluate recurrence-free survival based on patient characteristics analyzed according to a univariable and multivariable analyses

Variable	Univariable analysis			Multivariable analysis		
	HR	95% CI	*P* value	HR	95% CI	*P* value
Age (≤60 vs. ＞60)	1.12	0.59-2.12	0.7356			
Sex (male vs. female)	1.08	0.48-2.43	0.8522			
Operation (hepatectomy vs. liver transplantation)	0.75	0.33-1.70	0.4956			
Virus infection （yes vs. no）	1.04	0.32-3.37	0.9461			
Multiple nodules (yes vs. no)	4.98	2.61-9.51	<0.0001***	0.79	0.86-3.72	0.1216
MVI (yes vs. no)	8.7	4.36-17.36	<0.0001***	3	1.23-7.31	0.0157*
Thrombosis (yes vs. no)	4.7	2.51-8.80	<0.0001***	0.81	0.36-1.79	0.5961
AFP (≤20 vs. ＞20 ng/ml)	1.4	0.75-2.62	0.2855			
Differentiation (Poor vs. Good/moderate)	0.97	0.51-1.84	0.9156			
Tumor diameter (≤50 mm vs. ＞50 mm)	2.27	1.24-4.15	0.0081**	1.07	0.47-2.45	0.8659
CTCs number (≤15 vs. ＞15 /7.5ml)	11.89	5.00-28.30	<0.0001***	2.96	1.07-8.21	0.0375*
CTC cluster (yes vs. no)	13.97	7.00-27.88	<0.0001***	3.79	1.65-8.72	0.0017**
PCP （≤29.6% vs. ＞29.6%)	26.88	11.78-61.31	<0.0001***	13.18	5.01-34.67	<0.0001***

*, **, *** denote a P value of <0.05, <0.01 and <0.001 respectively. RFS, recurrence free survival; HCC, hepatocellular carcinoma; MVI, microvascular invasion; AFP, alpha-fetoprotein; CTC, circulating tumor cell; PCP, proliferative CTC percentage.
