# Supplementary material for: Tumor‐derived proliferative CTCs and CTC clusters predict aggressiveness and early recurrence in hepatocellular carcinoma patients
Source: Cancer Med. 2023 Jun 19;12(13):13912–27. doi: 10.1002/cam4.5946 (PMC10358265; doi:10.1002/cam4.5946)
Supplement: Supplementary file 2 — Figure S1–S6 [file CAM4-12-13912-s001.pdf]

Tumor-derived proliferative CTCs and CTC clusters predict aggressiveness and early recurrence in hepatocellular carcinoma patients

Supplementary Information

Supplementary Figures

Figure S1 The correlation and comparison between PCP and Ki67 expression in primary tumor.

Figure S2 Performance of driver gene mutation states in predicting recurrence-free survival.

Figure S3 Performance based on the status of mutations in 4 driver genes in CTCs for predicting recurrence-free survival.

Figure S4 Performance of CTC clusters in the training and validation data sets.

Figure S5 Performance of AFP level in predicting recurrence-free survival.

Figure S6 Performance of the nomogram predictor, CTC number, proliferative CTC percentage and CTC clusters in predicting recurrence-free survival.

Supplementary Tables

Table S1. Clinical and pathological characteristics of HCC patients

Table S2. Tumor tissue target sequencing panel

Table S3. Tumor mutations detected in target panel sequencing

Table S4. Tumor mutations detected in corresponding CTCs

Table S5. Correlation between clinical parameters and CTC number or proliferative CTC percentage in peripheral blood

Table S6. Cox regression model to evaluate recurrence-free survival based on patient characteristics analyzed according to a univariable and multivariable analysis

Supplementary Figure S1

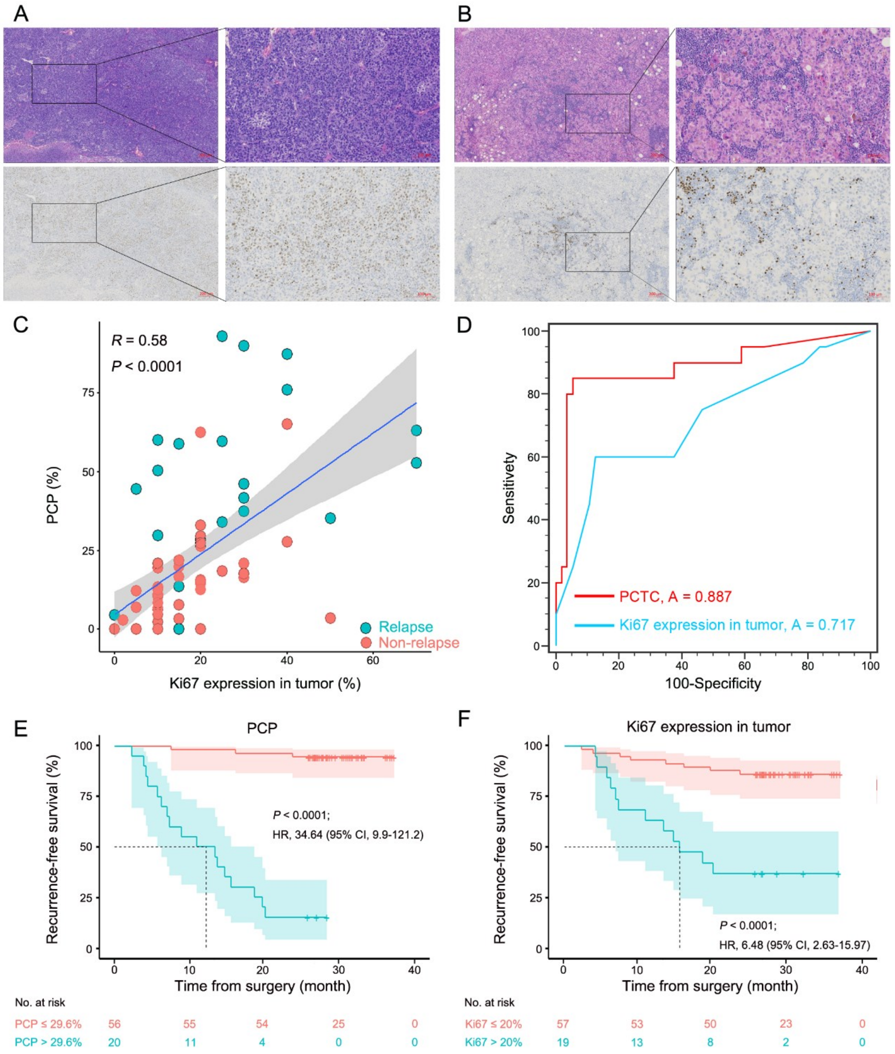

FIGURE S1 The correlation and comparison between PCP and Ki67 expression in primary tumor. FFPE tissue and hematoxylin and eosin (H&E) sections reviewed by two consultant pathologists. For immunohistochemical (IHC) staining, the tissue sections were deparaffinized in a series of gradient ethanol baths, rehydrated, and immersed in methanol containing 0.3% hydrogen peroxide for 10 min to block endogenous peroxidase at room temperature. Then, the tissue slides were heated for 30 min in a pH 6.0 antigen retrieval solution and then incubated overnight with either an anti-Ki67 antibody (Abcam, ab16667) or control immunoglobulin G (IgG, 1mg/mL) at 4°C. After washing with PBS, the slides were incubated with a Prolink-2 Plus HRP rabbit polymer detection kit (Golden Bridge, USA). The images were scanned using ZEISS Axioscan 7 (Carl Zeiss AG, Germany).

IHC of Ki67 in high (A) and low (B) Ki67 expression HCC patients. The correlation between PCP and Ki67 expression in primary tumor (n=76, person's r=0.58, $P < 0.0001$) (C). The capability of PCP and Ki67 expression in primary tumor in predicting HCC recurrence was estimated with ROC curves (D). Kaplan–Meier analysis of driver gene mutation states for predicting RFS in the PCP (E) and Ki67 expression in primary tumor (F). The cutoff value and P values were derived from log-rank (Mantel–Cox) tests, and HR values are indicated in the figure. The dashed lines in the figure represent the median survival (months). A, area under the curve; CI, confidence interval; CTC, criculating tumor cell; FFPE, formalin-fixed paraffin-embedded; HR, hazard ratio; PCP, proliferative CTC percentage; R, pearson correlation coefficient; RFS, recurrence-free survival.

Supplementary Figure S2

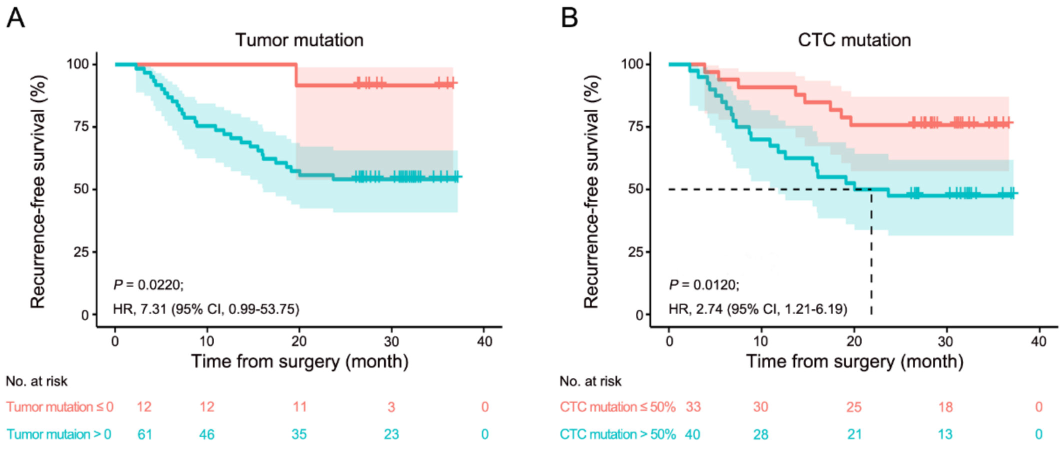

FIGURE S2 Performance of driver gene mutation states in predicting recurrence-free survival in tumor and CTC. Kaplan–Meier analysis of driver gene mutation status for predicting RFS in the (A) tumor tissue and (B) corresponding CTC. The cutoff value and *P* values were derived from log-rank (Mantel–Cox) tests, and HR values are indicated in the figure. The dashed lines in the figure represent the median survival (month). CI, confidence interval; CTC, circulating tumor cell; HR, hazard ratio; RFS, recurrence-free survival.

Supplementary Figure S3

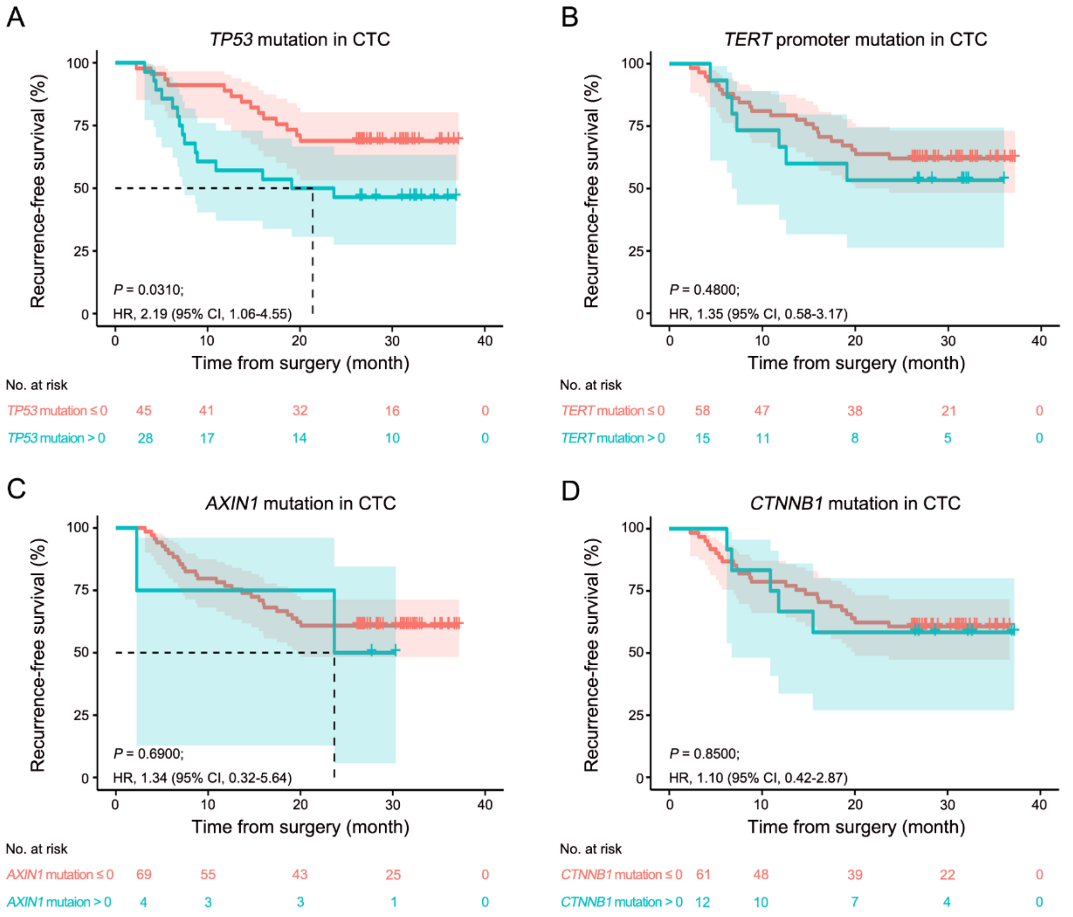

FIGURE S3 Performance based on the status of mutations in 4 driver genes in CTCs for predicting recurrence-free survival. Kaplan–Meier analysis of the mutation status of 4 driver genes, namely, *TP53* (A), *TERT* promoter (B), *AXIN1* (C) and *CTNNB1* (D), in CTCs for predicting RFS. The cutoff value and *P* values were derived from log-rank (Mantel–Cox) tests, and the HR values are indicated in the figure. The dashed lines in the figure represent the median survival (month). CI, confidence interval; CTC, circulating tumor cell; HR, hazard ratio; RFS, recurrence-free survival.

Supplementary Figure S4

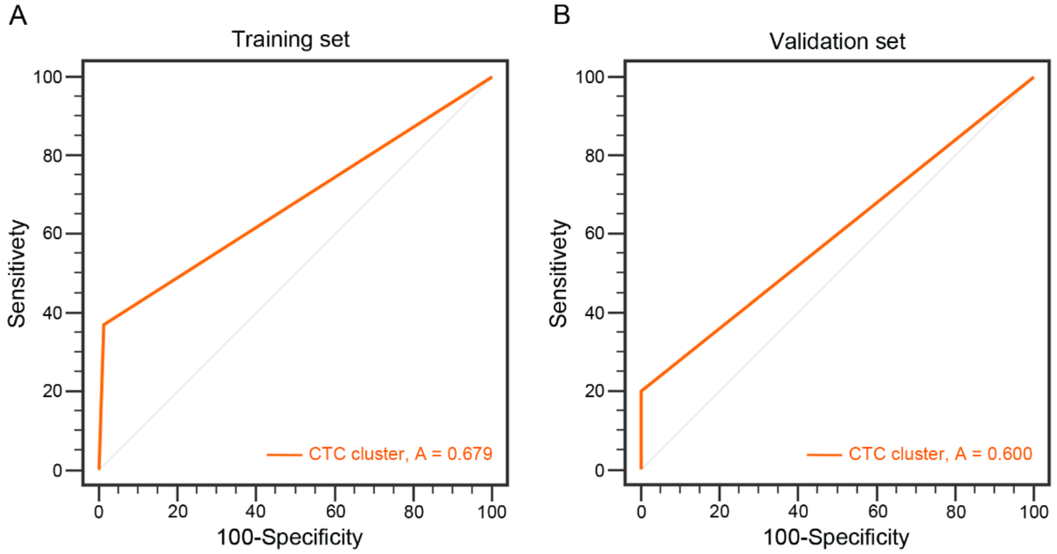

C

Training set

	Relapse	Non-relapse	
Predicted relapse	10	1	
Predicted non-relapse	17	76	
Totals	27	77	Totals
Correct	10	76	86
Sensitivity (%)	37.04		
Specificity (%)		98.70	

Predict accuracy：82.69%

D

Validation set

	Relapse	Non-relapse	
Predicted relapse	3	0	
Predicted non-relapse	12	37	
Totals	15	37	Totals
Correct	3	37	40
Sensitivity (%)	20.00		
Specificity (%)		100.00	

Predict accuracy：76.92%

FIGURE S4 Performance of CTC clusters in the training and validation data sets. (A) and (B) The capability of CTC clusters in predicting HCC recurrence was estimated with ROC curves in the training data set and validation data set. (C) and (D) Confusion tables of the binary results of CTC clusters in the training and validation data sets. A, area under the curve; CTC, circulating tumor cell.

Supplementary Figure S5

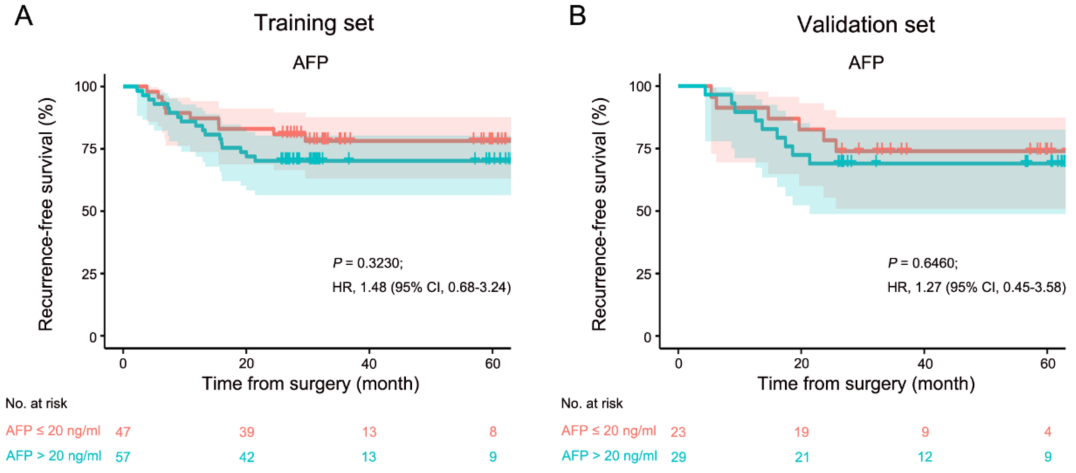

FIGURE S5 Performance of AFP in predicting recurrence-free survival. Kaplan–Meier analysis of AFP for predicting RFS in the (A) training data set and (B) validation data set. The cutoff value and *P* values were derived from log-rank (Mantel–Cox) tests, and HR values are indicated in the figure. The dashed lines in the figure represent the median survival (month). AFP, alpha-fetoprotein; CI, confidence interval; HR, hazard ratio; RFS, recurrence-free survival.

Supplementary Figure S6

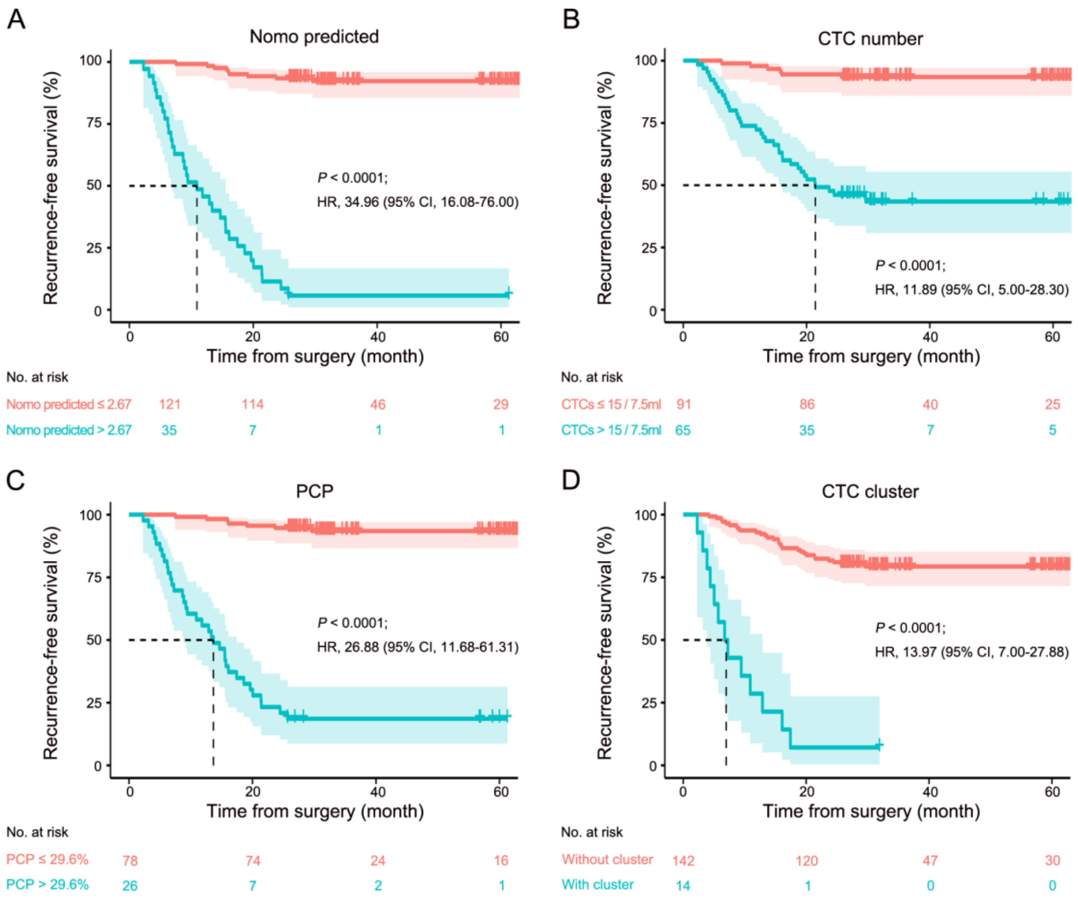

FIGURE S6 Performance of the nomogram predictor, CTC number, proliferative CTC percentage and CTC clusters in predicting recurrence-free survival. Kaplan–Meier analysis of (A) nomogram predictor, (B) CTC number, (C) PCP and (D) CTC clusters for predicting RFS in the total data set. The cutoff value and *P* values were derived from log-rank (Mantel–Cox) tests, and HR values are indicated in the figure. The dashed lines in the figure represent the median survival (month). CI, confidence interval; CTC, circulating tumor cell; HR, hazard ratio; PCP, proliferative CTC percentage; RFS, recurrence-free survival.
